# Supplementary material for: Structural and mechanistic characterization of bifunctional heparan sulfate N-deacetylase-N-sulfotransferase 1
Source: Nat Commun. 2024 Feb 13;15:1326. doi: 10.1038/s41467-024-45419-4 (PMC10864358; doi:10.1038/s41467-024-45419-4)
Supplement: Supplementary file 1 — Supplementary Information [file 41467_2024_45419_MOESM1_ESM.pdf]

Supplementary information for

Structural and mechanistic characterization of bifunctional heparan sulfate N-deacetylase-N-sulfotransferase 1

Courtney J. Mycroft-West[1], Sahar Abdelkarim[1], Helen M. E. Duyvesteyn[2], Neha S. Gandhi[3,4,5], Mark A. Skidmore[6], Raymond J. Owens[1,2], Liang Wu[1,2]

1 – The Rosalind Franklin Institute, Harwell Science & Innovation Campus, Didcot, OX11 0QX, U.K.

2 – Division of Structural Biology, Nuffield Department of Medicine, University of Oxford, The Wellcome Centre for Human Genetics, Oxford, OX3 7BN, U.K.

3 – Centre for Genomics and Personalised Health, Queensland University of Technology, Brisbane, QLD 4059, Australia

4 – School of Chemistry and Physics, Queensland University of Technology, Brisbane, QLD 4000, Australia

5 – Department of Computer Science and Engineering, Manipal Institute of Technology, Manipal Academy of Higher Education, Manipal, UDUPI, Karnataka-576104, India

6 – Centre for Glycoscience Research and Training, Keele University, Newcastle-Under-Lyme, ST5 5BG, U.K.

Correspondence to liang.wu@rfi.ac.uk

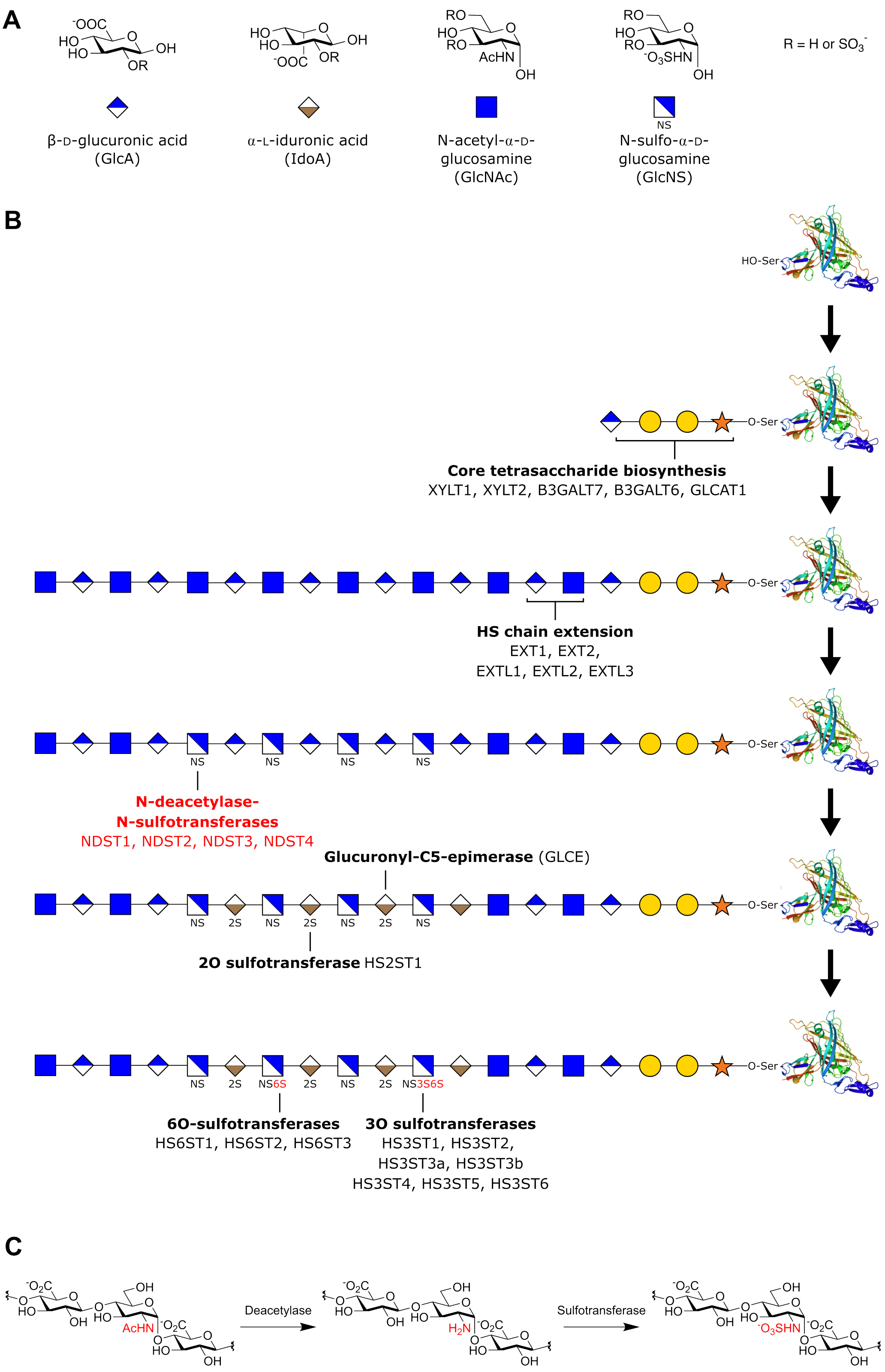

Supplementary Figure 1 Chemical structure and biosynthesis pathway of heparan sulfate (HS). (A) Monosaccharide building blocks involved in HS construction. Sites of variable O-sulfation are annotated. (B) Canonical biosynthesis pathway of HS within the Golgi complex. NDST enzymes act after chain polymerization by the EXT enzymes. (C) Bifunctionality of NDST enzymes – GlcNAc sugars are first deacetylated to glucosamine by catalytic deacetylase activity, before N-sulfation by catalytic sulfotransferase activity.

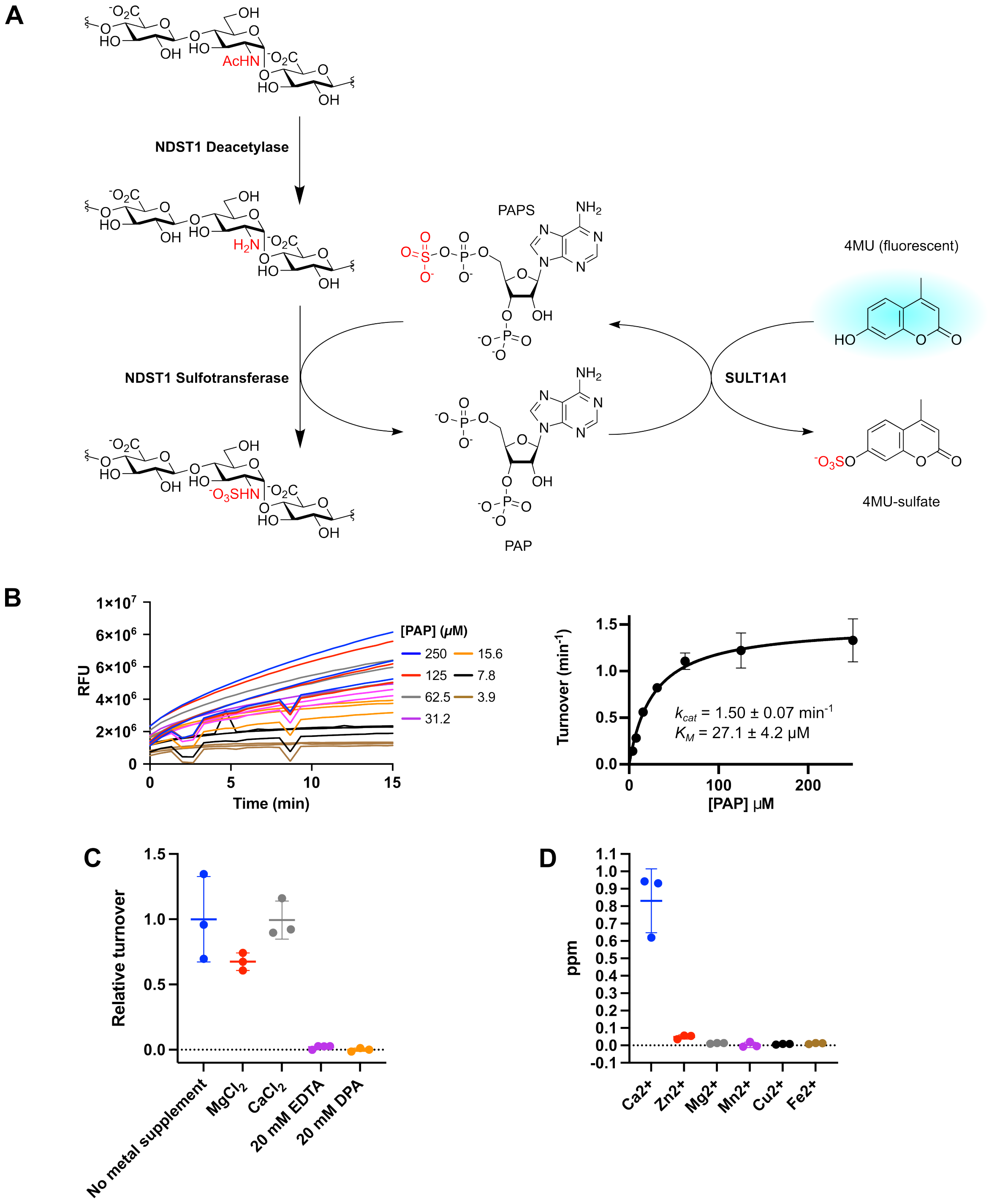

Supplementary Figure 2 Coupled enzyme assay to measure NDST1 activity. (A) Schematic of assay: 5'-phosphoadenosine-3'-phosphate (PAP) generated by NDST1 is regenerated by the bacterial enzyme Sult1A1, using fluorogenic sulfate donor 4-methylumbelliferyl (4MU) sulfate. (B) Representative time course measurement of Sult1A1 mediated 4MU release with respect to PAP concentration (left), Michaelis-Menten kinetics of Sult1A1 activity with respect to PAP (right). Datapoints show mean ± s.d. from N = 3 technical replicates run on the same assay plate. Uncertainties in kinetic constants represent standard error values from curve fitting. RFU – relative fluorescence units. (C) Relative activity of NDST1 in the presence of Mg^{2+}, Ca^{2+} and chelators, as measured using the Sult1A1 coupled enzyme assay. (D) ICP-OES of purified NDST1, showing the presence of carried through Ca^{2+} and (trace) Zn^{2+}. Datapoints are plotted from N = 3 technical replicates. Bars show mean ± s.d. Ppm – parts per million. Source data for graphs are provided within the Source Data file.

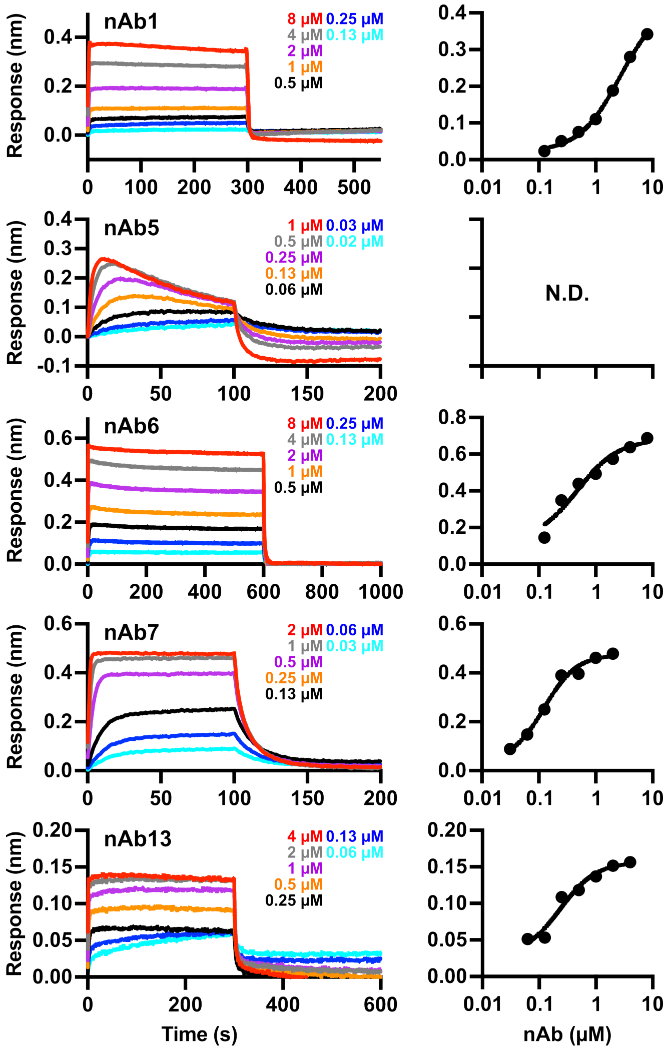

Supplementary Figure 3 Biophysical characterization of anti-NDST1 nAb affinities by biolayer interferometry (BLI). Representative sensorgrams and steady state binding curves showing interaction of nAbs with bound NDST1. Quantitated binding parameters are shown in main text **Table 1**. N.D. not determinable due to poor curve fit. Measurements were made in singlicate N = 1. Source data for BLI traces and binding curves are provided within the Source Data file.

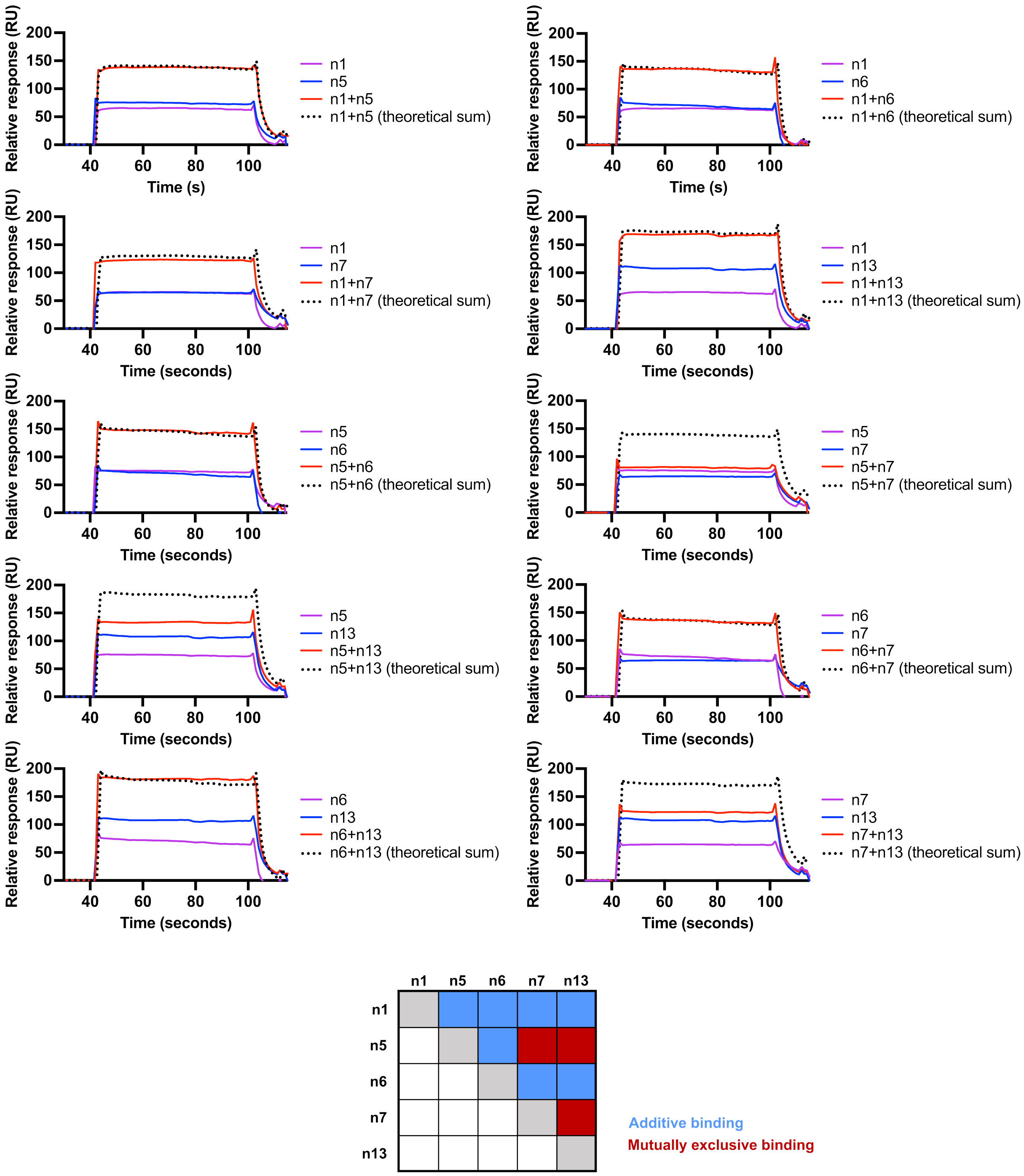

Supplementary Figure 4 Additive binding of anti-NDST1 nAbs, as measured by SPR of combinatorial nAb pairs. Binding of nAb5+nAb7, nAb5+nAb13 or nAb7+nAb13 is mutually exclusive. RU – response units. SPR traces are representative plots from N = 3 technical replicates – all showed similar results. Source data for all traces are provided within the Source Data file.

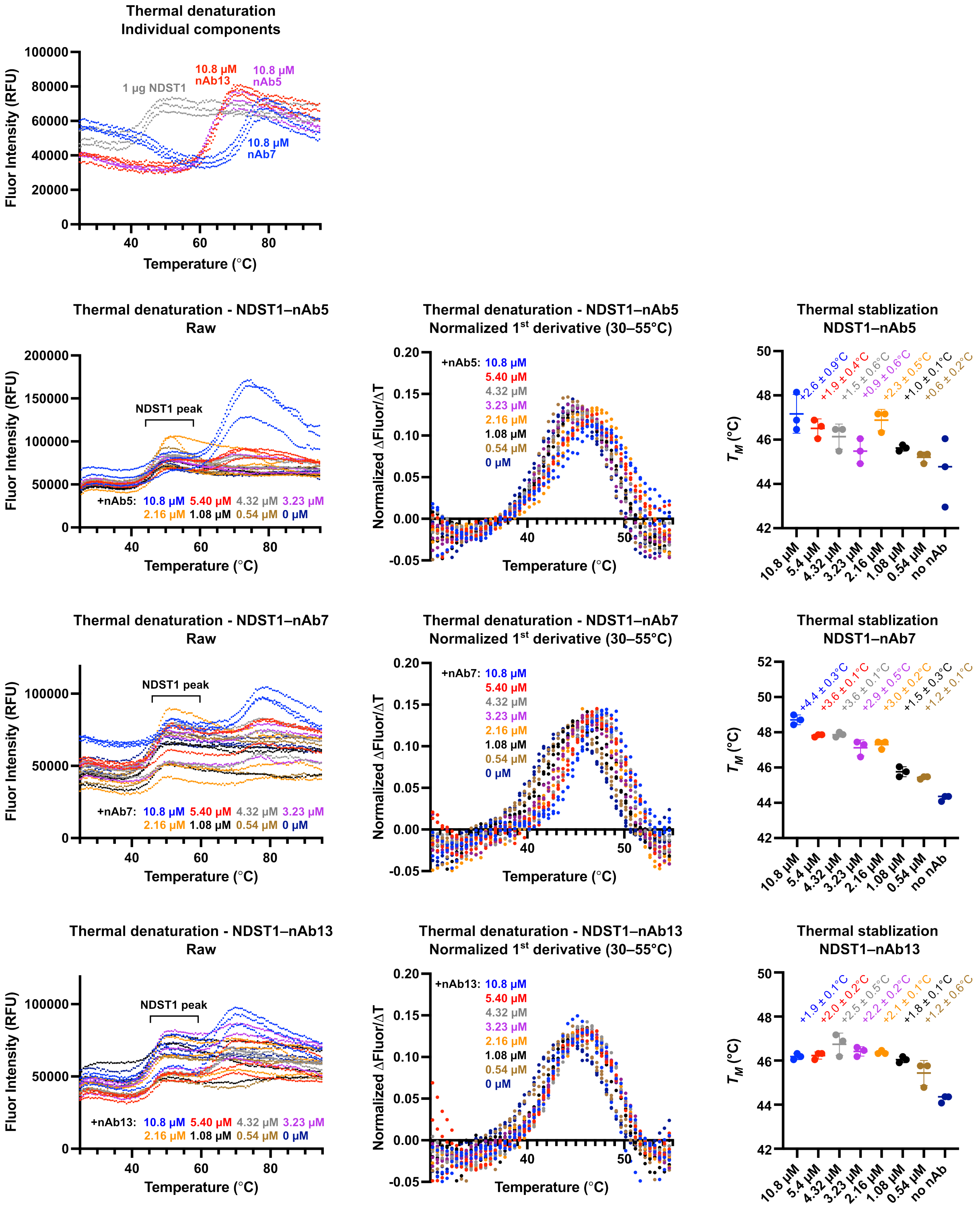

Supplementary Figure 5 Differential scanning fluorimetry showing thermal stabilization of NDST1 in the presence of nAb7 and nAb13. Datapoints for all graphs are individual measurements. Denaturation temperature (T_M) was calculated from peaks of the first derivative of the normalized melting curves for NDST1 (30–55°C). Bars in thermal stabilization plots are mean ± s.d. from N = 3 technical replicates run on the same assay plate. RFU – relative fluorescence units. Source data for all graphs are provided within the Source Data file.

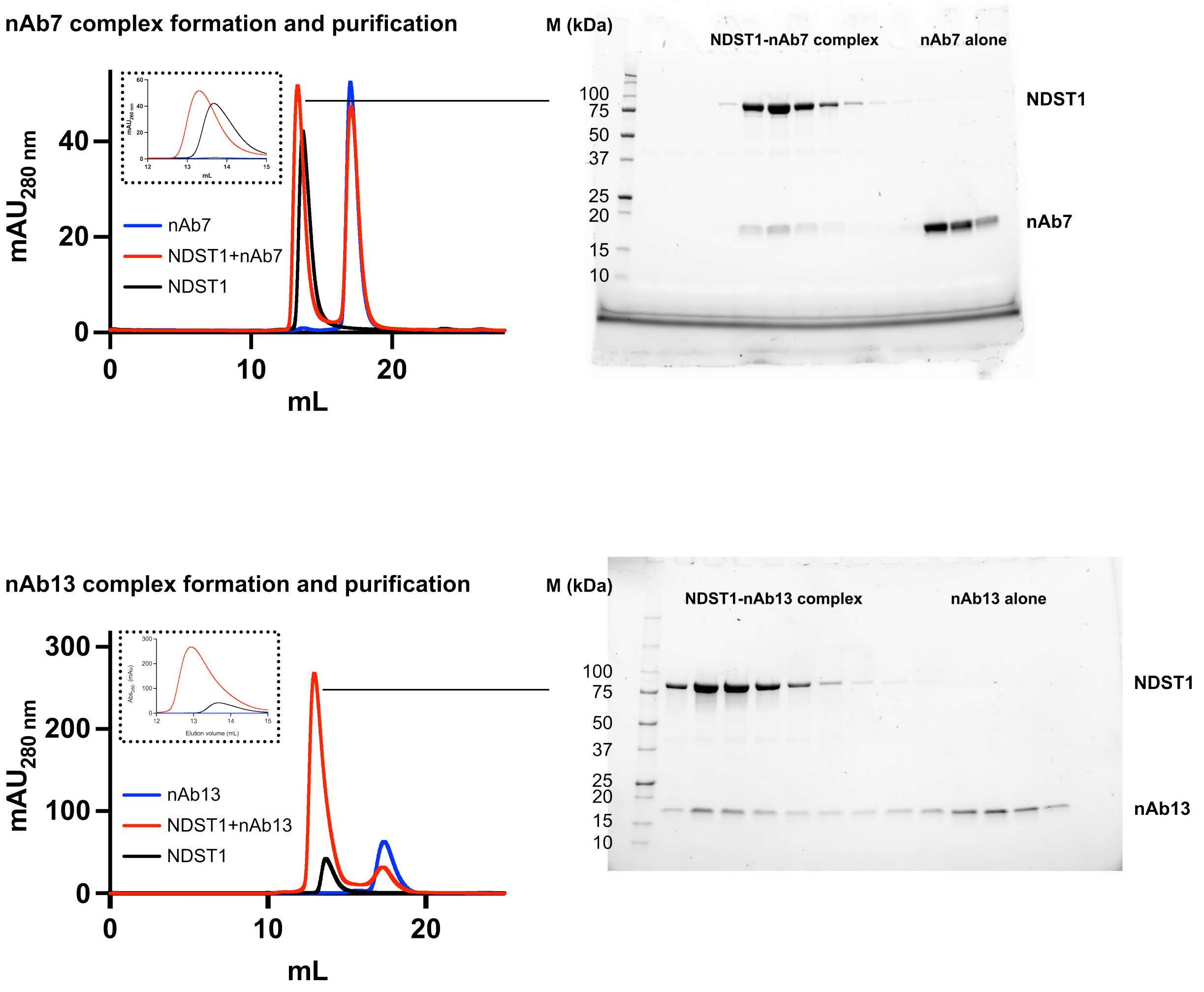

Supplementary Figure 6 SEC purification of NDST1 in complex with nAbs. nAb7 or nAb13 binding induces a shift in NDST1 retention time, indicative of stable complex formation. SDS-PAGE gel confirms co-elution of NDST1 with nAbs. $mAU_{280\ nm}$ – milli-absorbance units at λ = 280 nm.

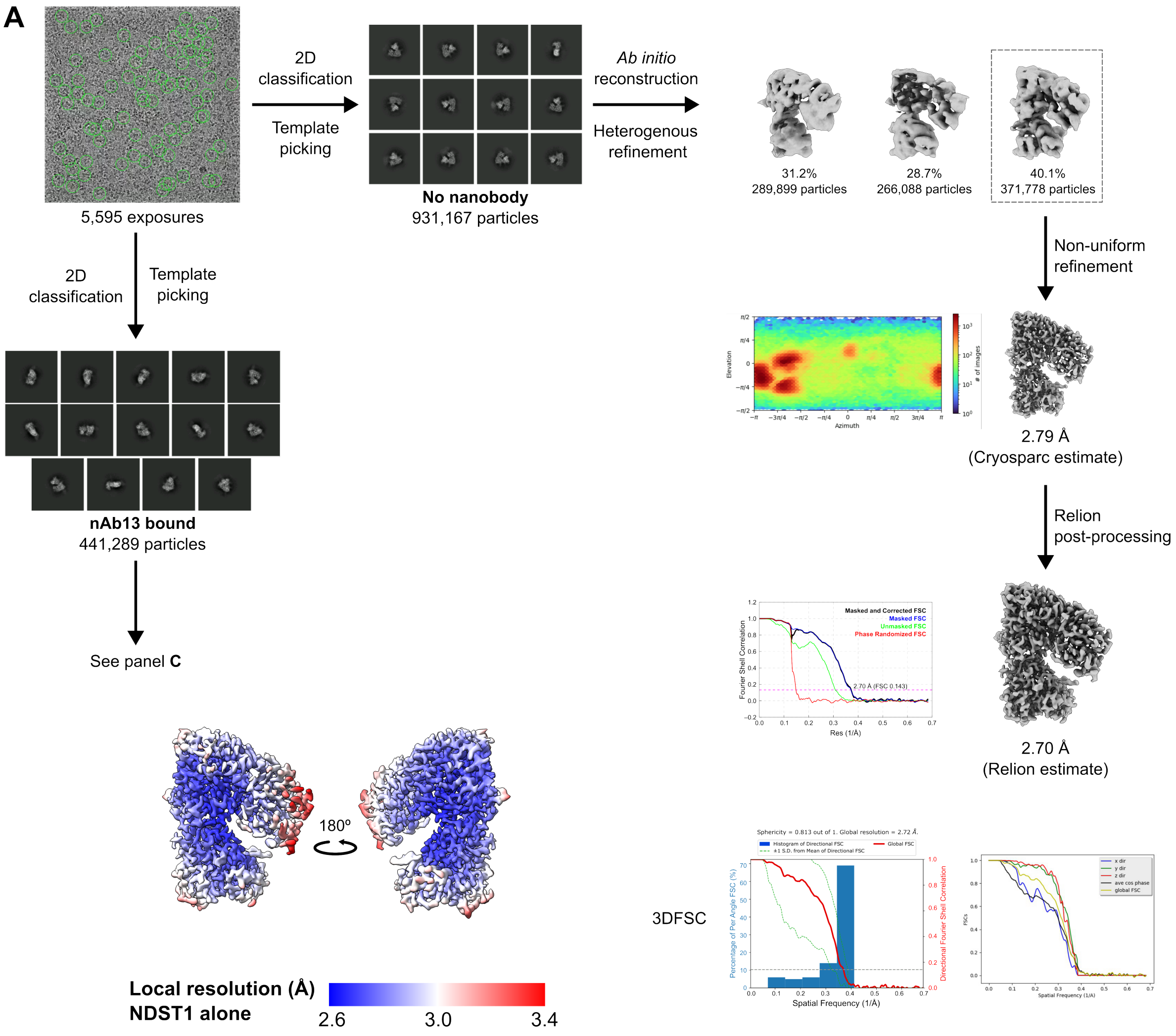
A
5,595 exposures
2D classification
Template picking
No nanobody
931,167 particles
Ab initio reconstruction
Heterogenous refinement
31.2%
289,899 particles
28.7%
266,088 particles
40.1%
371,778 particles
Non-uniform refinement
2.79 Å
(Cryosparc estimate)
Relion post-processing
2.70 Å
(Relion estimate)
3DFSC
2D classification
Template picking
nAb13 bound
441,289 particles
See panel C
180º
Local resolution (Å)
NDST1 alone
2.6
3.0
3.4
Masked and Corrected FSC
Masked FSC
Unmasked FSC
Phase Randomized FSC
2.70 Å (FSC 0.143)
Fourier Shell Correlation
Res (1/Å)
Elevation
Azimuth
Spatial Frequency (1/Å)

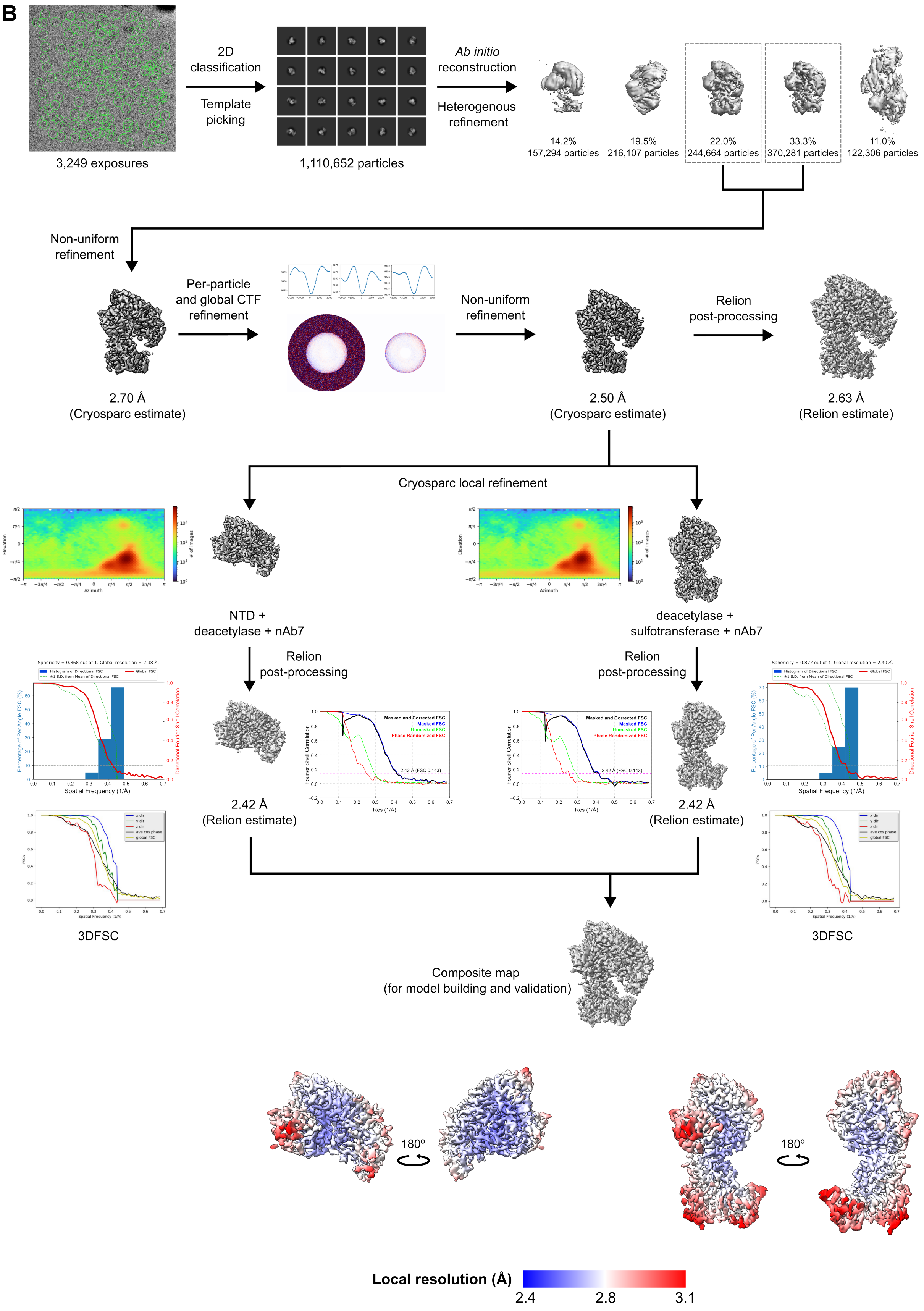

B
3,249 exposures
2D classification
Template picking
1,110,652 particles
Ab initio reconstruction
Heterogenous refinement
14.2%
157,294 particles
19.5%
216,107 particles
22.0%
244,664 particles
33.3%
370,281 particles
11.0%
122,306 particles
Non-uniform refinement
Per-particle and global CTF refinement
Non-uniform refinement
Relion post-processing
2.70 Å
(Cryosparc estimate)
2.50 Å
(Cryosparc estimate)
2.63 Å
(Relion estimate)
Cryosparc local refinement
NTD + deacetylase + nAb7
deacetylase + sulfotransferase + nAb7
Relion post-processing
Relion post-processing
2.42 Å
(Relion estimate)
2.42 Å
(Relion estimate)
Masked and Corrected FSC
Masked FSC
Unmasked FSC
Phase Randomized FSC
Fourier Shell Correlation
Res (1/Å)
Sphericity = 0.868 out of 1. Global resolution = 2.38 Å.
Sphericity = 0.877 out of 1. Global resolution = 2.40 Å.
Histogram of Directional FSC
Global FSC
Percentage of Per Angle FSC (%)
Directional Fourier Shell Correlation
Spatial Frequency (1/Å)
Elevation
Azimuth
# of images
3DFSC
3DFSC
Composite map
(for model building and validation)
180º
180º
Local resolution (Å)
2.4
2.8
3.1

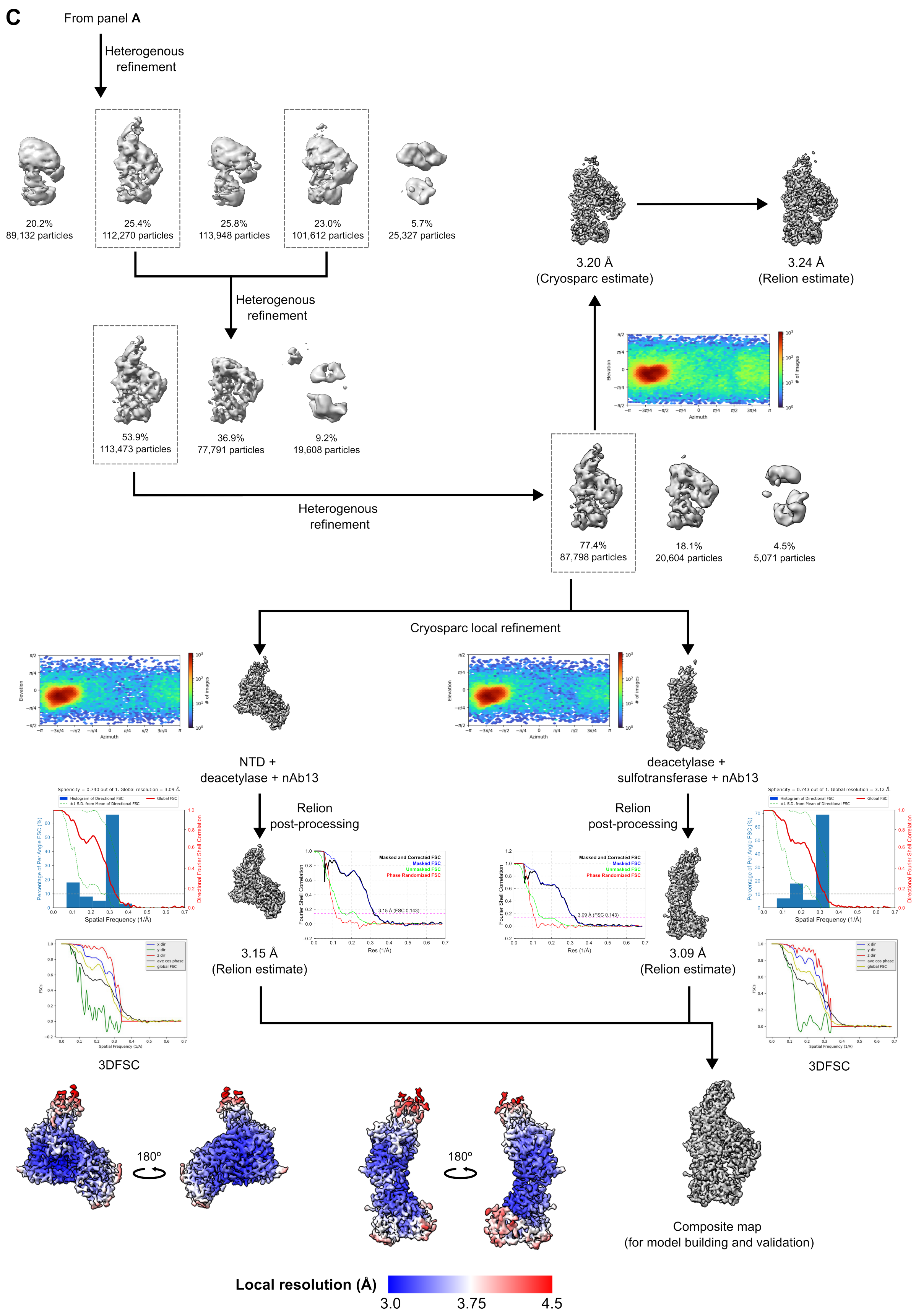
C
From panel A
Heterogenous refinement
20.2%
89,132 particles
25.4%
112,270 particles
25.8%
113,948 particles
23.0%
101,612 particles
5.7%
25,327 particles
Heterogenous refinement
53.9%
113,473 particles
36.9%
77,791 particles
9.2%
19,608 particles
Heterogenous refinement
77.4%
87,798 particles
18.1%
20,604 particles
4.5%
5,071 particles
3.20 Å
(Cryosparc estimate)
3.24 Å
(Relion estimate)
Cryosparc local refinement
NTD +
deacetylase + nAb13
deacetylase +
sulfotransferase + nAb13
Relion
post-processing
Relion
post-processing
3.15 Å
(Relion estimate)
3.09 Å
(Relion estimate)
3DFSC
3DFSC
180º
180º
Composite map
(for model building and validation)
Local resolution (Å)
3.0
3.75
4.5

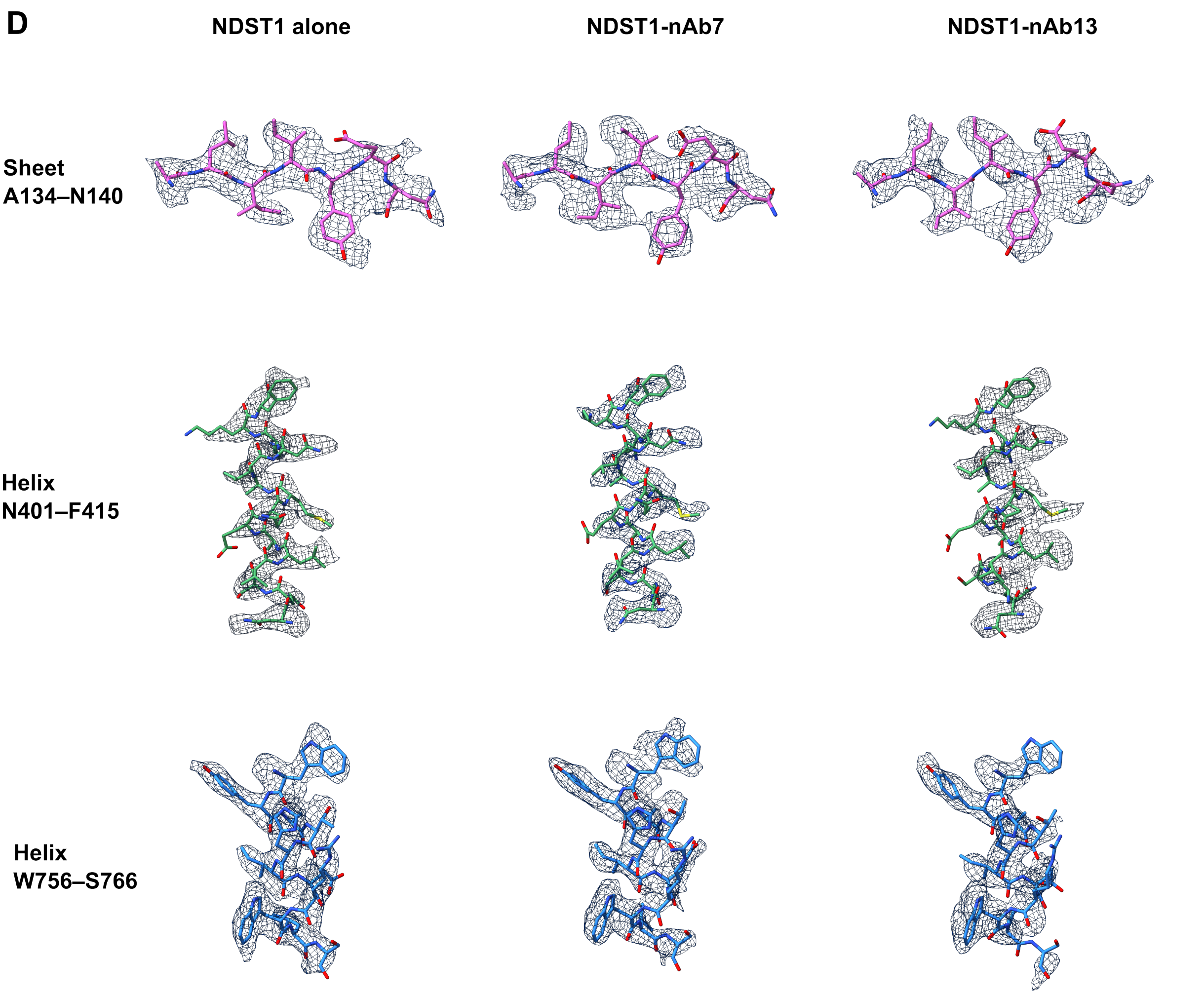

Supplementary Figure 7 Cryo-EM processing strategies and data quality. (A) nAb free NDST1. (B) NDST1-nAb7 complex. (C) NDST1-nAb13 complex. Note that nAb free NDST1 and NDST1-nAb13 volumes were reconstructed from particles on the same grid. (D) Representative densities and model fits for structures. Maps are contoured to ChimeraX 0.12 for A134–N140, ChimeraX 0.18–0.24 for N401–F415, ChimeraX 0.14–0.15 for W756–S766.

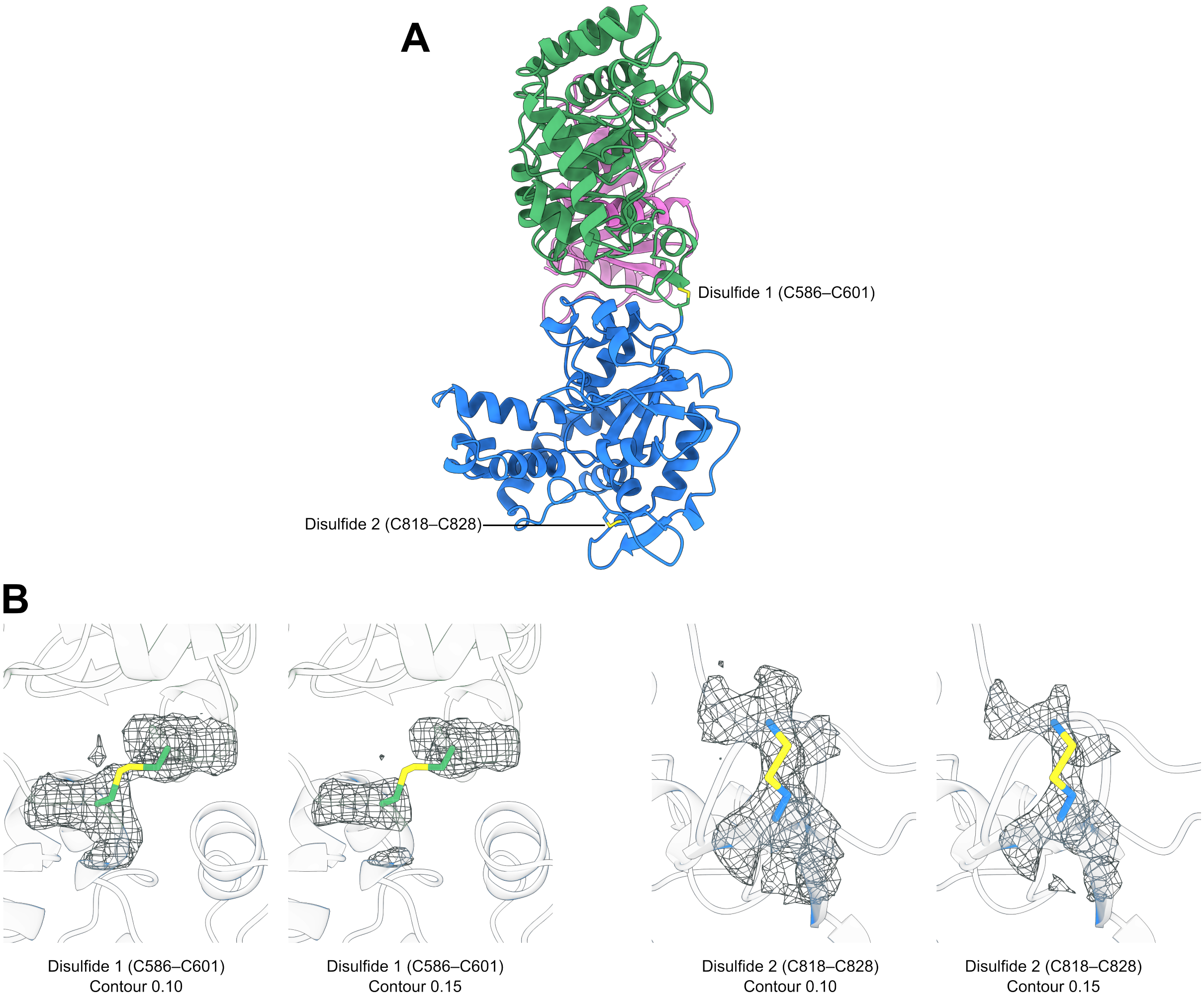

Supplementary Figure 8 NDST1 disulfide bonds. (A) Locations of disulfide bond 1 (C586–C601) and disulfide bond 2 (C818–C828) within the context of the NDST1 structure. Disulfide 1 occupies a solvent exposed position at the interface between the NDST1 deacetylase and sulfotransferase domains. (B) Coulombic density within 3 Å of NDST1 disulfide bond cystines, contoured at different levels in ChimeraX. Density for disulfide 1 is only apparent at low contours, consistent with reduced occupancy compared to disulfide 2.

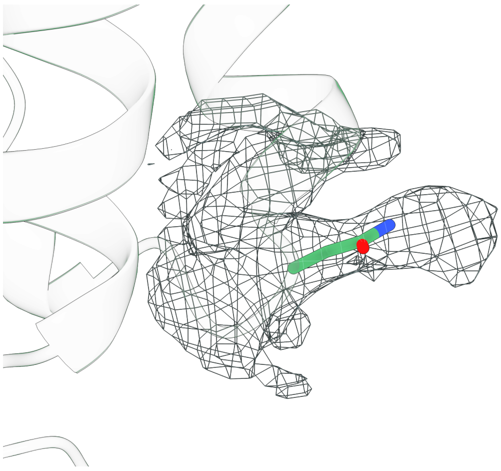

Supplementary Figure 9 Additional density consistent with the first GlcNAc of an N-glycan tree near the sidechain of N401.

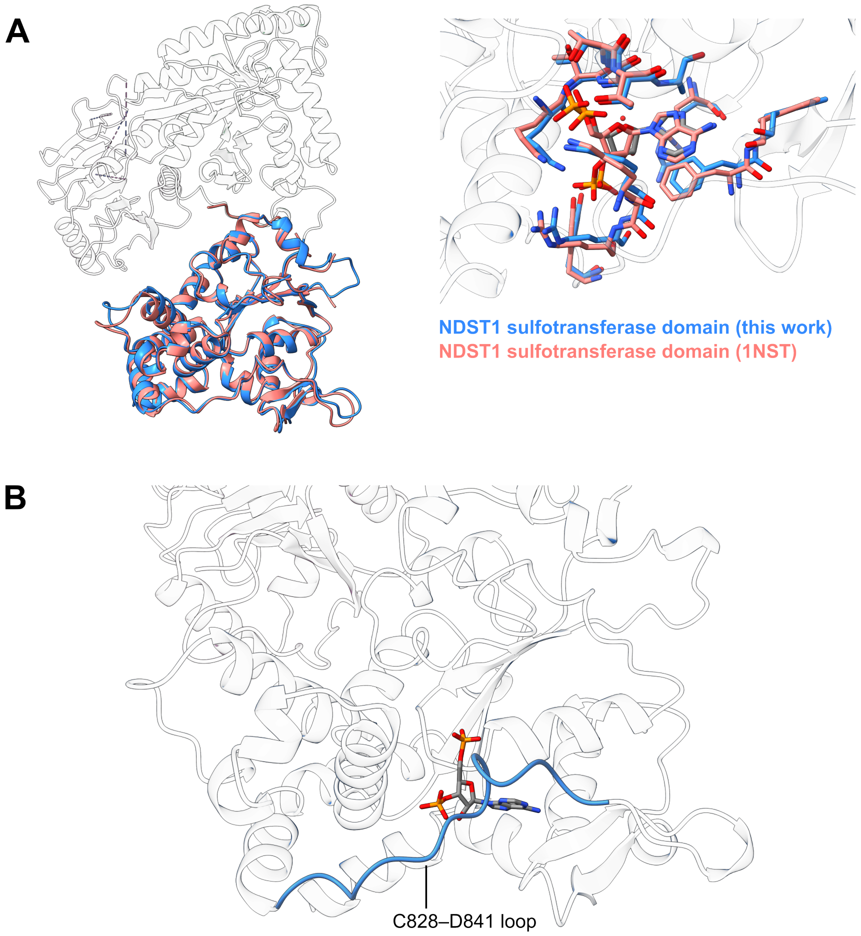

Supplementary Figure 10 Additional views of NDST1 sulfotransferase domain. (A) Comparison of NDST1 sulfotransferase domain overall fold and active site architecture with previously solved crystal structure 1NST. RMSD 0.96 Å over 277 Cαs. (B) Location of the C828–D841 loop, which appears to function as a lid that holds PAP(S) within the sulfotransferase active site.

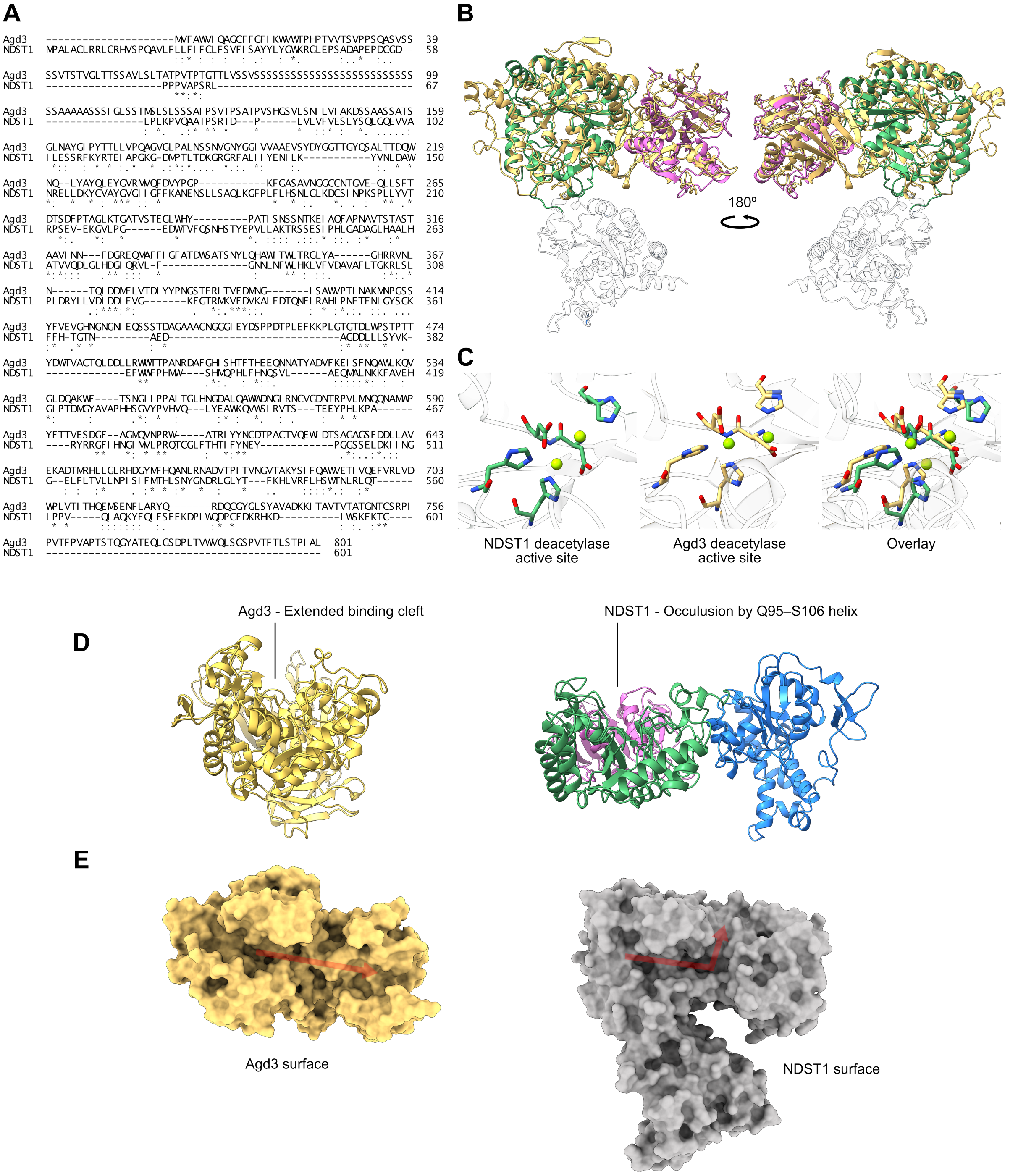

Supplementary Figure 11 NDST1 homology with Agd3 from *Aspergillus fumigatus* (PDB accession 6NWZ). (A) ClustalΩ[1] alignment of Agd3 with NDST1 residues 1-601 (N-terminal and deacetylase domains) showing 21.5% sequence identity. (B) Superposition of Agd3 and NDST1, showing similarity over N-terminal and deacetylase domains. NDST1 colored as in **Main Text Figure 3**, Agd3 colored in yellow. (C) Deacetylase domain active site comparisons for NDST1 and Agd3. Two divalent metal ions are observed close to the catalytic amino acids for both enzymes. (D) Agd3 binds substrates using an extended cleft, spanning its deacetylase domain and carbohydrate binding accessory domain. (E) A similar extended cleft in NDST1 is occluded by the NTD Q95-S106 helix. However, a binding channel can be observed at the interface between the deacetylase and N-terminal domains.

A

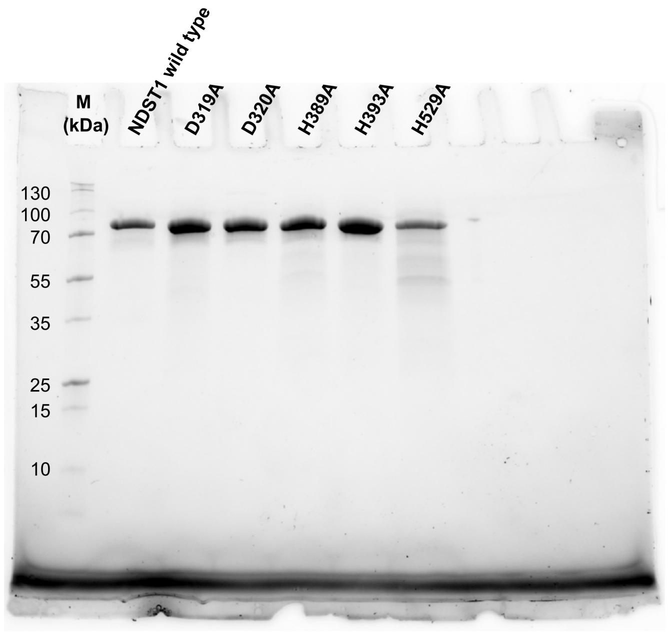

B

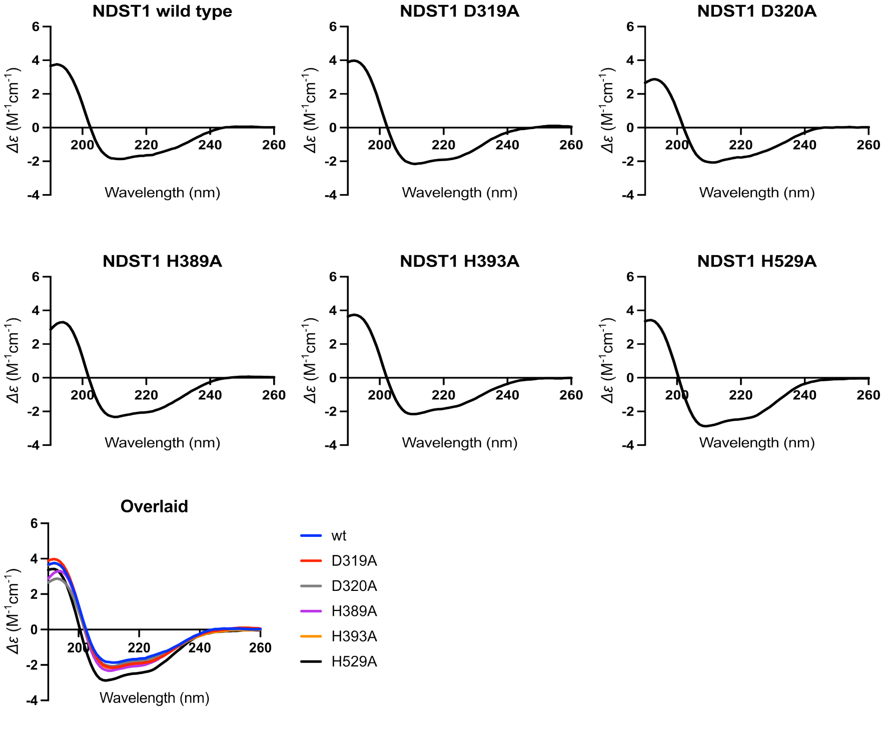

Supplementary Figure 12 SDS-PAGE (A) and circular dichroism spectra (B) indicate similar molecular mass and secondary structure composition for wild-type NDST1 vs deacetylase domain mutants. *Δε* – molar circular dichroism at given wavelength. Each CD trace was measured in singlicate (N = 1). Source data for all CD traces are provided within the Source Data file.

A

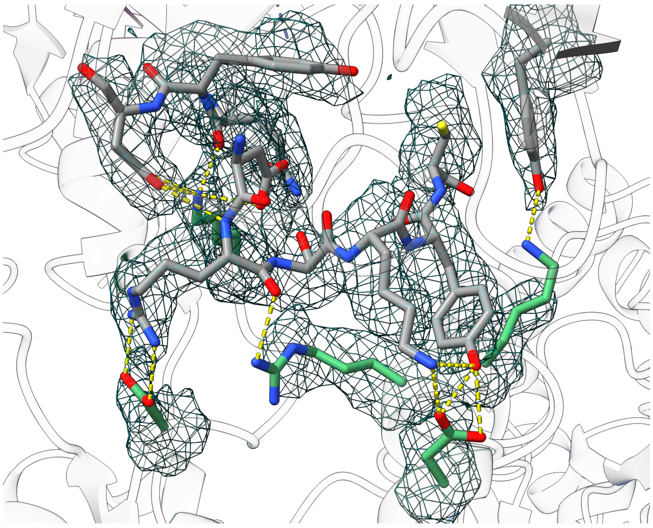

B

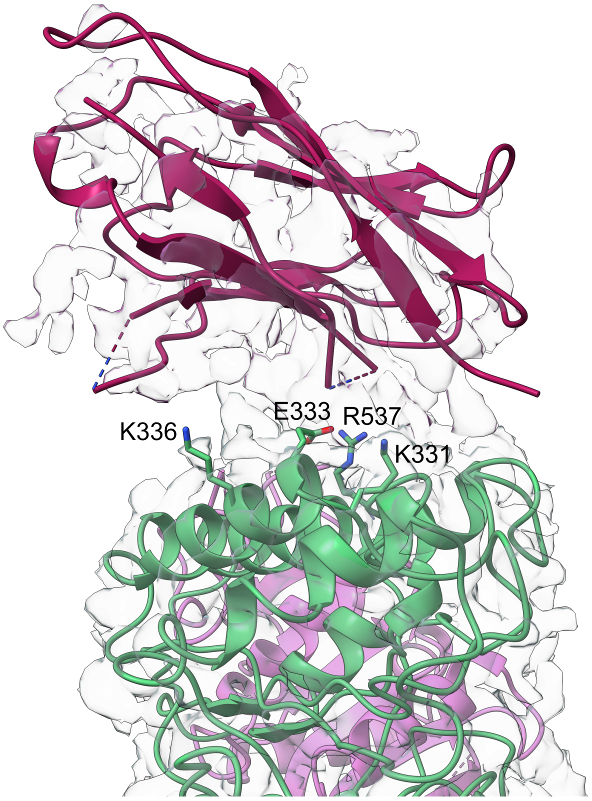

Supplementary Figure 13 cryo-EM maps of NDST1 nAb binding interfaces. (A) NDST1-nAb7 interface, with coulombic density contoured to 0.15 in ChimeraX. View is identical to that in **Main Text Figure 3c**. (B) NDST1-nAb13 interface. NDST1 residues within 6 Å of the rigid body fitted nAb13 (K331, E333, K336 and R537) are annotated. Coulombic density contoured to 0.12 in ChimeraX.

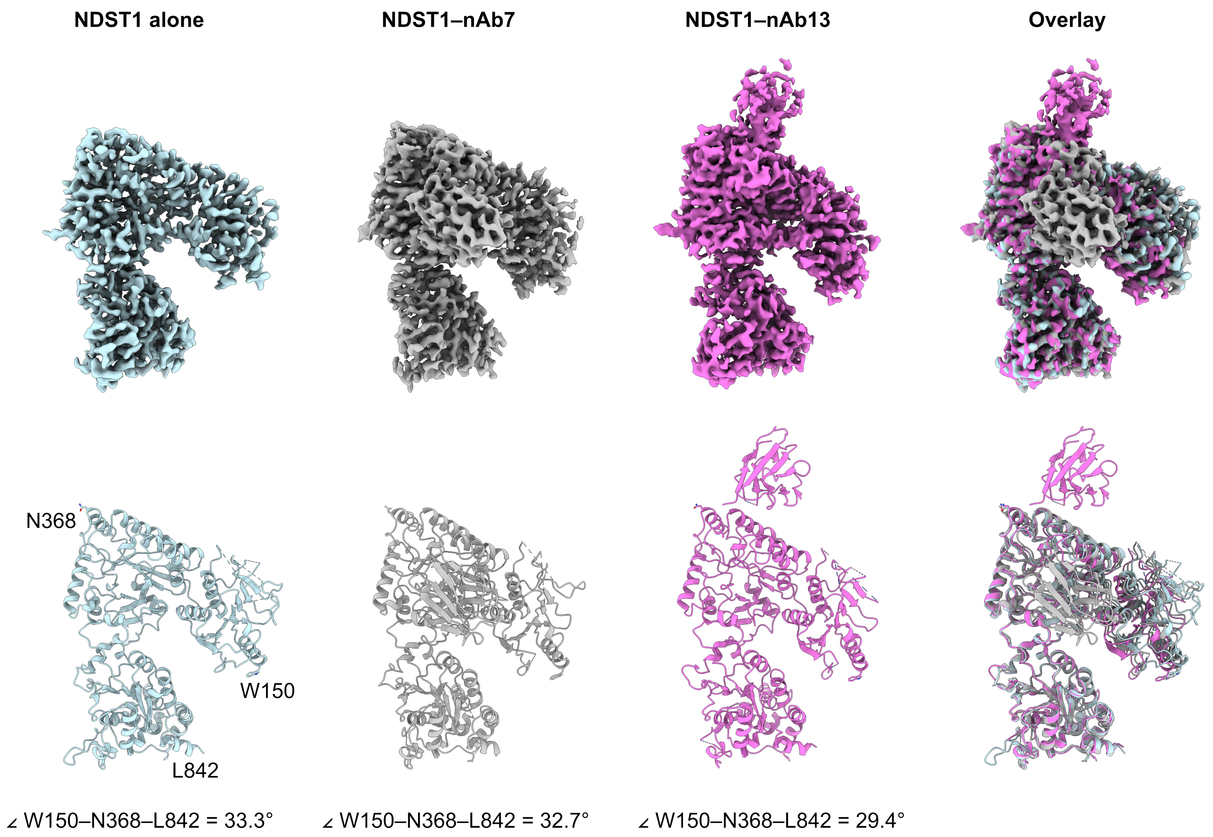

Supplementary Figure 14 Hinge-like movements of the NDST1 structure. The NDST1-nAb7 and NDST1-nAb13 complexes display a modest contraction of the NDST1 elbow compared to nAb free NDST1, as quantitated by the angle between W150–N368–L842 (Cαs). Densities are contoured to 0.15, 0.18 and 0.15 in ChimeraX for NDST1 alone, NDST1-nAb7, and NDST1-nAb13 volumes respectively.

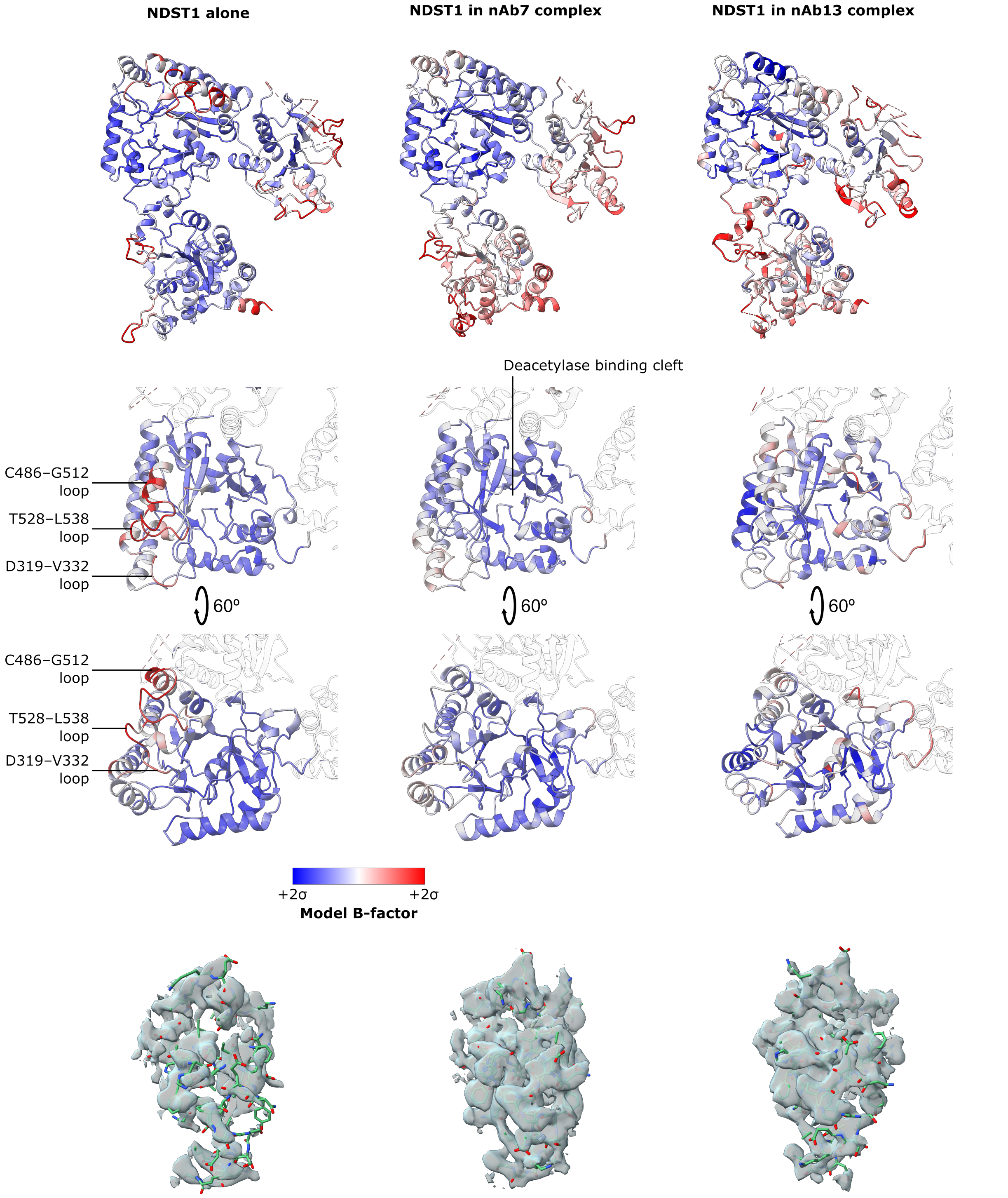

Supplementary Figure 15 B-factor analysis of NDST1 models from the NDST1 alone, NDST1-nAb7 and NDST1-nAb13 datasets. Higher B-factors (red) indicate increased structural disorder and molecular movement. Both nAb7 and nAb13 induce ordering of the D319–V332, C486–G512 and T528–L538 loops, which lie close to the deacetylase cleft, and are likely to contact substrate. Comparison of map volume around the D319–V332, C486–G512 and T528–L538 region shows nAb free NDST1 to have weaker density, indicating poor ordering of these loops. All model B factors are normalized to mean ± 2 s.d. Maps contoured to 0.12 in ChimeraX for all structures.

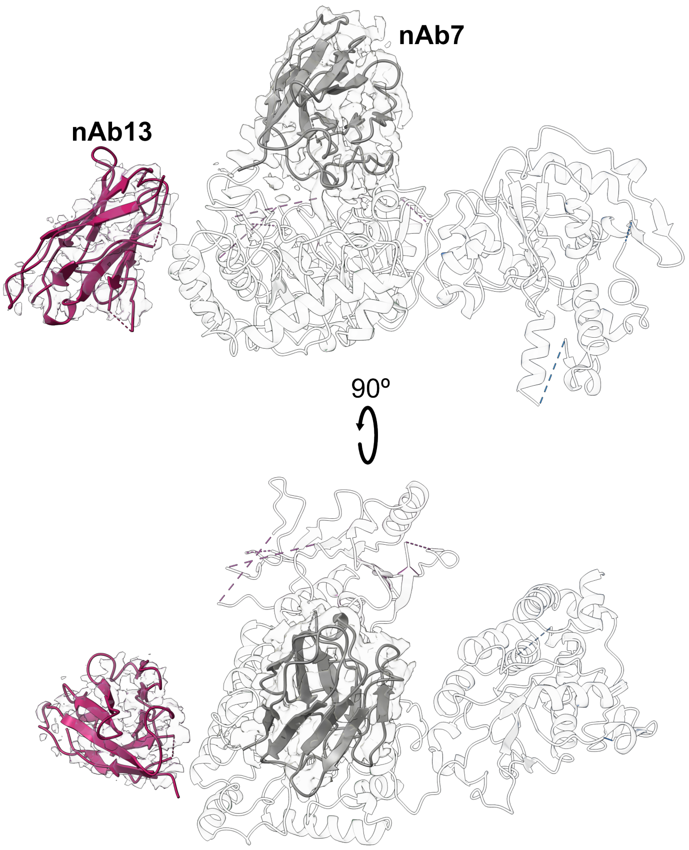

Supplementary Figure 16 Overlay of nAb7 and nAb13 binding sites on NDST1. No direct steric interference is observed between the two nAbs, suggesting their inability to mutually bind NDST1 (**Supplementary Figure 4**) arises from allosteric effects.

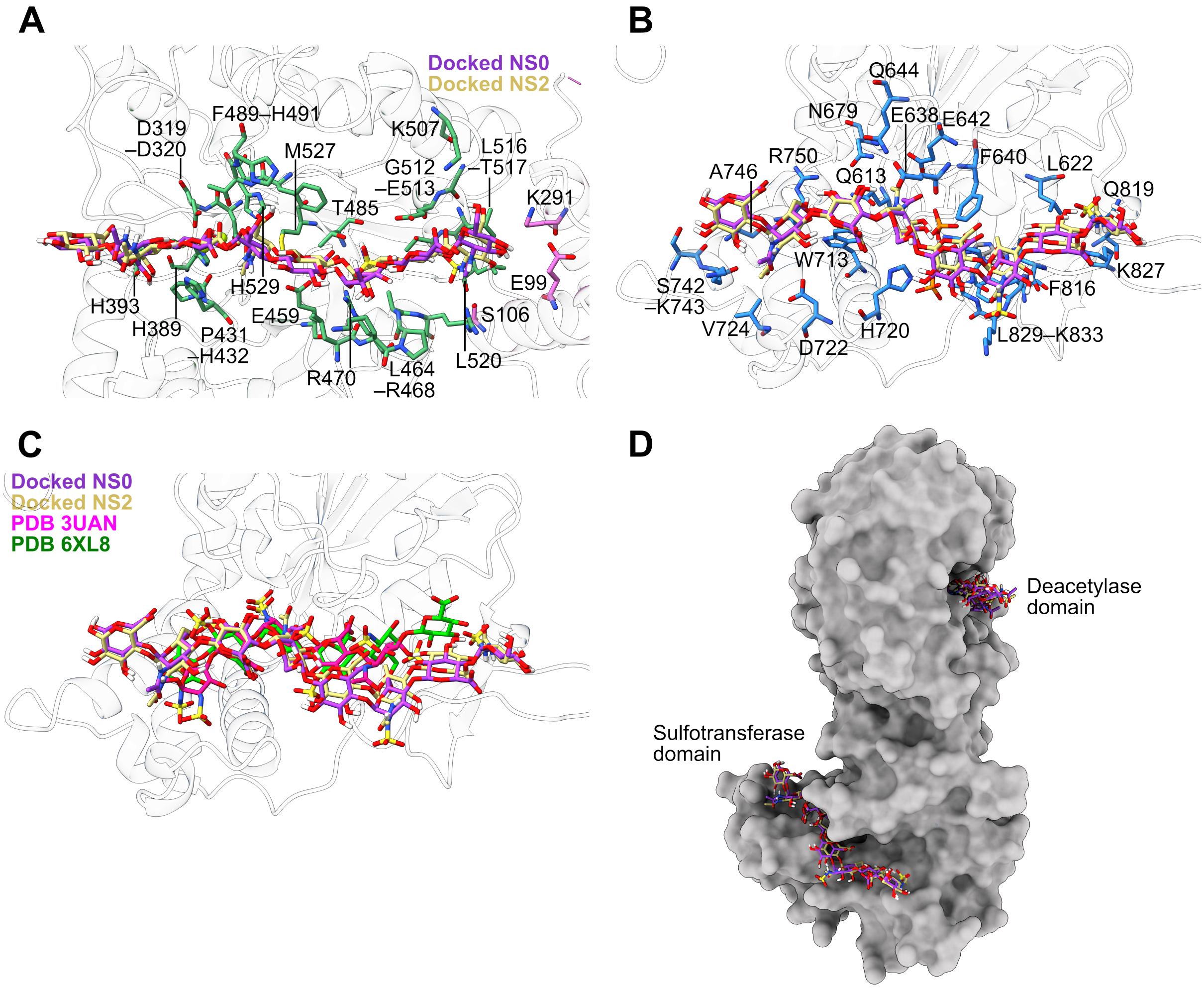

Supplementary Figure 17 Close up views of oligosaccharides docked into NDST1 active sites. (A) NS0 (purple) and NS2 (yellow) in the NDST1 deacetylase cleft, with all amino acids within 4 Å annotated. Residues from both deacetylase and N-terminal domains are present at the interaction interface. (B) NS0 and NS2 in the NDST1 sulfotransferase cleft, with active site amino acids and amino acids within 4 Å annotated. (C) Comparison of docked NS0 and NS2 poses in the NDST1 sulfotransferase cleft with structures of homologous sulfotransferases HS3ST1 (3UAN) and HS3ST3 (6XL8). Trajectories of the NDST1 docked octasaccharides are similar to those experimentally observed for HS3ST1 and HS3ST3. (D) Surface overview showing the spatial relationship between oligosaccharides in the NDST1 deacetylase and sulfotransferase domains.

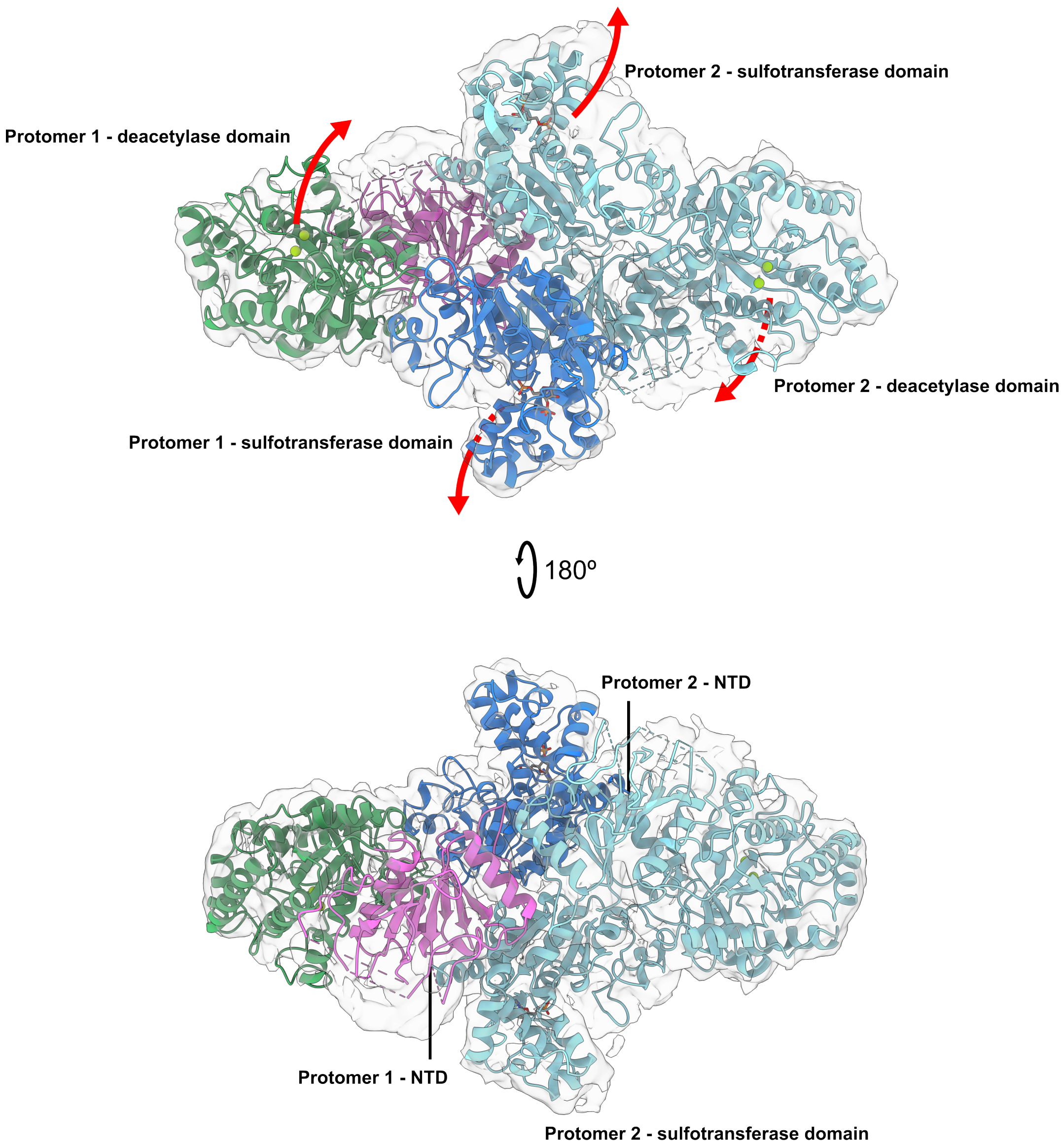

Supplementary Figure 18 Dimeric NDST1. Docking of our nAb free NDST1 model into the dimeric volume reported by Vallet et al[2]. Protomer 1 is colored according to **Main Text Figure 3**, protomer 2 is pale blue. (Top view) The dimeric interface creates a new arrangement of NDST1 catalytic domains, between the sulfotransferase domain of one protomer and the deacetylase domain of a neighbor. Red arrows highlight the orientations of each catalytic domain in the NDST1 homodimer. (Bottom view) Dimerization interface formed between the NTD of one NDST1 protomer, and the NTD and sulfotransferase domain of its partner.

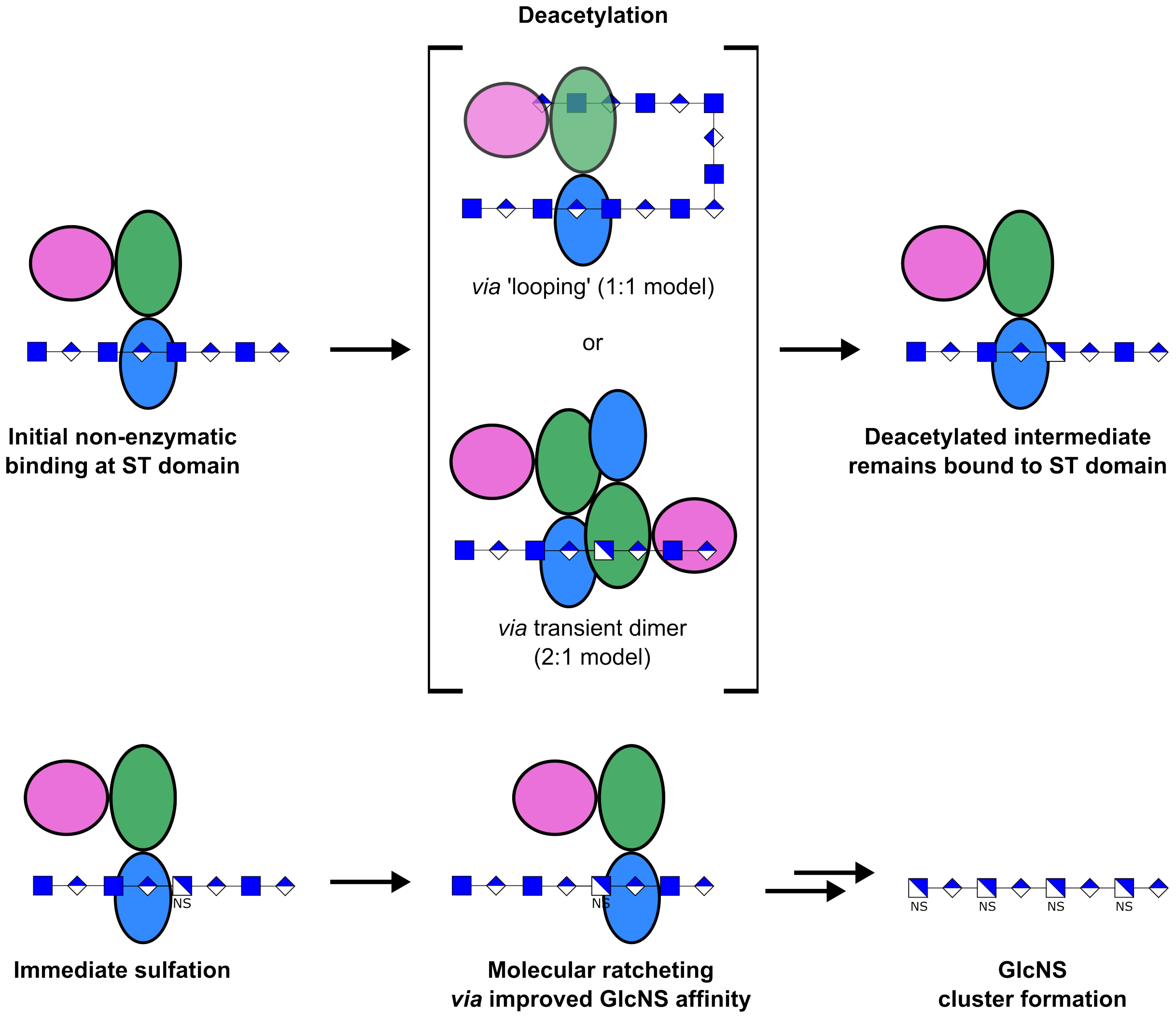

Supplementary Figure 19 Putative model for bifunctional NDST1 catalysis. Initial HS binding at the NDST1 sulfotransferase domain occurs prior to enzymatic processing. Deacetylation, either by looping or transient dimerization, leads to the formation of a GlcN intermediate, which is immediately processed by the vicinal sulfotransferase active site. Because sulfotransferase domain interactions are electrostatic, they may interact more strongly with GlcNS residues, producing a ratcheting effect that can drive GlcNS cluster formation. Domain colors correspond to those in **Main Text Figure 3**.

Supplementary Table 1 Cryo-EM data processing and model building statistics

	NDST1 alone map and model **(EMD-16564) (PDB 8CCY)**	NDST1-nAb7 NTD–DeAc–nAb7 original map **(EMD-16626)**	NDST1-nAb7 NTD–DeAc–nAb7 locally refined map **(EMD-16627)**	NDST1-nAb7 DeAc–ST–nAb7 locally refined map **(EMD-16629)**	NDST1-nAb7 Composite map and model **(EMD-16565) (PDB 8CD0)**	NDST1-nAb13 NTD–DeAc–nAb13 original map **(EMD-16661)**	NDST1-nAb13 NTD–DeAc–nAb13 locally refined map **(EMD-16662)**	NDST1-nAb13 DeAc–ST–nAb13 locally refined map **(EMD-16663)**	NDST1-nAb13 Composite map and model **(EMD-16664) (PDB 8CHS)**
**Data collection and processing**									
Magnification	165,000x	165,000x	165,000x	165,000x		165,000x	165,000x	165,000x	
Voltage (kV)	300	300	300	300		300	300	300	
Electron exposure (e–/Å^2)	50	50	50	50		50	50	50	
Defocus range (μm)	-1.2 to -2.6	-1.2 to -2.6	-1.2 to -2.6	-1.2 to -2.6		-1.2 to -2.6	-1.2 to -2.6	-1.2 to -2.6	
Pixel size (Å)	0.73	0.73	0.73	0.73		0.73	0.73	0.73	
Symmetry imposed	C1	C1	C1	C1		C1	C1	C1	
Initial particle images (no.)	3,867,804	2,721,982	2,721,982	2,721,982		3,867,804	3,867,804	3,867,804	
Final particle images (no.)	371,778	614,945	614,945	614,945		87,798	87,798	87,798	
Map resolution (Å)	2.70	2.64	2.42	2.42		3.24	3.15	3.09	
FSC threshold	0.143	0.143	0.143	0.143		0.143	0.143	0.143	
Map resolution range (Å)	2.66–4.58	2.55–4.74	2.53–3.45	2.58–3.49		3.06–8.44	3.01–5.70	3.14–6.27	
**Refinement**									
Initial model used (PDB code)	Alphafold2 prediction				NDST1 alone model; 7TGF				NDST1 alone model;<br>nAb7 (CA only)
Model resolution (Å)	2.9				3.2				4.0
FSC threshold	0.5				0.5				0.5
Map sharpening *B* factor (Å^2)	0				0				0
Model composition									
Non-hydrogen atoms	6235				6860				6424
Protein residues	754				837				820
Ligands	3				2				2
B factors (Å^2)									
Protein	85.5				88.8				62.1
Ligand	72.6				133.2				77.9
R.m.s. deviations									
Bond lengths (Å)	0.003				0.007				0.008
Bond angles (°)	0.561				1.25				1.65
Validation									
MolProbity score	1.59				1.62				1.95
Clashscore	3.01				3.48				5.94
Poor rotamers (%)	3.29				2.03				2.19
Ramachandran plot									
Favored (%)	97.45				96.34				94.64
Allowed (%)	2.55				3.66				5.36
Disallowed (%)	0.00				0.00				0.00

Supplementary Table 2 NDST1 and nanobody sequences

Construct	ORF DNA sequence	Protein sequence
NDST (79–882)	ATGAAATTTTTGGTGAACGTGGCCTTGGTGTTTATGGTGGTTTACATTAGCTACATTTATGC<br>GGACCCGGGCCATCATCACCACCATCATGGTACCGCAGAAAACTTGTACTTTCAAGGCTCCC<br>GCACAGACCCGTTGGTGCTGGTCTTTGTGGAGAGCCTCTACTCGCAACTGGGCCAGGAGGTG<br>GTGGCCATCCTGGAGTCCAGCCGCTTCAAATACCGCACAGAGATTGCGCCGGGCAAGGGTGA<br>CATGCCCACGCTCACTGACAAGGGCCGTGGCCGCTTCGCCCTCATCATCTATGAGAACATCC<br>TCAAGTATGTCAACCTGGACGCCTGGAACCGGGAGCTGCTGGACAAGTACTGTGTGGCCTAC<br>GGCGTGGGCATCATTGGCTTCTTCAAGGCCAATGAGAACAGCCTGCTGAGTGCGCAGCTCAA<br>GGGCTTCCCCCTGTTCCTGCACTCAAACCTGGGCCTGAAGGACTGCAGCATCAACCCCAAGT<br>CCCCGCTGCTCTACGTGACGCGACCTAGCGAGGTGGAGAAAGGTGTGCTCCCCGGCGAGGAC<br>TGGACGGTTTTCCAGTCAAATCACTCCACCTATGAGCCAGTGCTGCTGGCCAAGACGCGCTC<br>GTCAGAGTCCATCCCACACCTGGGCGCAGACGCCGGCCTGCATGCTGCACTGCACGCCACTG<br>TGGTCCAGGACCTGGGCCTGCACGACGGCATCCAGCGCGTGCTGTTTGGCAACAACCTGAAC<br>TTCTGGCTGCACAAGCTTGTCTTCGTGGATGCCGTGGCCTTCCTCACGGGGAAGCGCCTCTC<br>CCTGCCATTGGACCGCTACATCCTGGTGGACATTGATGACATCTTCGTGGGCAAGGAGGGCA<br>CACGCATGAAGGTGGAGGACGTGAAGGCCCTGTTTGACACACAGAACGAACTACGCGCACAC<br>ATCCCAAACTTCACCTTCAACCTGGGCTACTCAGGGAAATTCTTCCACACAGGTACCAATGC<br>TGAGGACGCTGGGGATGATCTGCTGCTGTCGTATGTGAAGGAGTTCTGGTGGTTCCCCCACA<br>TGTGGAGCCACATGCAGCCCCACCTTTTCCACAACCAGTCCGTGTTGGCCGAGCAGATGGCC<br>TTGAACAAGAAGTTCGCTGTCGAGCATGGCATTCCCACAGACATGGGGTATGCAGTGGCGCC<br>CCACCACTCGGGCGTGTACCCCGTGCACGTGCAGCTGTACGAGGCTTGGAAGCAAGTGTGGA<br>GCATCCGCGTGACCAGCACGGAGGAGTACCCCCACCTGAAGCCAGCCCGCTACCGCCGTGGC<br>TTCATCCACAATGGCATCATGGTTCTCCCACGGCAGACCTGCGGCCTCTTCACACACACCAT<br>CTTCTACAACGAGTACCCTGGCGGCTCCAGTGAGCTGGACAAGATCATCAACGGGGGCGAGC<br>TCTTCCTCACCGTGCTCCTCAATCCTATCAGCATCTTCATGACGCACCTGTCCAACTATGGG<br>AATGACCGCCTGGGCCTGTACACCTTCAAGCACCTGGTGCGCTTCCTGCACTCCTGGACGAA<br>CCTCCGGCTGCAGACACTGCCCCCTGTGCAGTTGGCGCAGAAGTACTTCCAGATCTTCTCCG<br>AGGAGAAGGACCCGCTCTGGCAGGACCCCTGCGAGGACAAACGTCACAAAGACATCTGGTCC<br>AAGGAGAAGACGTGTGACCGCTTCCCAAAGCTCCTCATCATCGGCCCCCAGAAAACAGGCAC<br>CACTGCCCTCTACCTGTTCCTGGGCATGCACCCTGACCTAAGCAGCAACTACCCCAGCTCTG<br>AGACATTTGAGGAGATCCAGTTTTTTAATGGCCACAACTATCACAAAGGCATCGACTGGTAC<br>ATGGAGTTCTTCCCCATCCCTTCCAACACCACGTCCGACTTCTACTTTGAGAAAAGCGCCAA<br>CTACTTTGATTCAGAAGTGGCGCCCCGGCGGGCAGCAGCCCTCTTGCCCAAAGCCAAGGTCC<br>TGACCATCCTCATCAACCCCGCGGACCGGGCCTATTCCTGGTACCAGCACCAGCGAGCCCAT<br>GACGACCCAGTGGCCCTAAAGTACACCTTCCATGAGGTGATTACCGCCGGCTCTGACGCATC<br>CTCGAAGCTGCGTGCCCTCCAGAACCGCTGCCTGGTCCCTGGCTGGTACGCCACCCACATCG<br>AGCGCTGGCTCAGTGCCTATCACGCCAACCAGATTCTGGTCTTGGATGGCAAACTGCTTCGC<br>ACAGAACCTGCCAAAGTGATGGACATGGTGCAGAAGTTCCTTGGGGTGACCAACACCATTGA<br>CTACCACAAAACCTTGGCGTTTGATCCAAAGAAAGGATTTTGGTGCCAACTGCTTGAAGGAG<br>GAAAAACCAAGTGTCTGGGCAAAAGCAAGGGCCGGAAATATCCCGAGATGGACTTGGATTCC<br>CGAGCCTTCCTGAAGGACTATTACCGGGACCACAACATCGAGCTCTCCAAGCTGCTGTATAA<br>GATGGGCCAGACACTTCCCACTTGGCTACGAGAGGACCTCCAGAACACCAGGTAA	MKFLVNVALVFMVVYISYIYADPGHHHHHHGTAENLYFQGSRTDPLVLVFVESLYSQLGQEVVA<br>ILESSRFKYRTEIAPGKGDMPTLTDKGRGRFALIIYENILKYVNLDAWNRELLDKYCVAYGVGI<br>IGFFKANENSLLSAQLKGFPLFLHSNLGLKDCSINPKSPLLYVTRPSEVEKGVLPGEDWTVFQS<br>NHSTYEPVLLAKTRSSESIPHLGADAGLHAALHATVVQDLGLHDGIQRVLFGNNLNFWLHKLVF<br>VDAVAFLTGKRLSLPLDRYILVDIDDIFVGKEGTRMKVEDVKALFDTQNELRAHIPNFTFNLGY<br>SGKFFHTGTNAEDAGDDLLLSYVKEFWWFPHMWSHMQPHLFHNQSVLAEQMALNKKFAVEHGIP<br>TDMGYAVAPHHSGVYPVHVQLYEAWKQVWSIRVTSTEEYPHLKPARYRRGFIHNGIMVLPRQTC<br>GLFTHTIFYNEYPGGSSELDKIINGGELFLTVLLNPISIFMTHLSNYGNDRLGLYTFKHLVRFL<br>HSWTNLRLQTLPPVQLAQKYFQIFSEEKDPLWQDPCEDKRHKDIWSKEKTCDRFPKLLIIGPQK<br>TGTTALYLFLGMHPDLSSNYPSSETFEEIQFFNGHNYHKGIDWYMEFFPIPSNTTSDFYFEKSA<br>NYFDSEVAPRRAAALLPKAKVLTILINPADRAYSWYQHQRAHDDPVALKYTFHEVITAGSDASS<br>KLRALQNRCLVPGWYATHIERWLSAYHANQILVLDGKLLRTEPAKVMDMVQKFLGVTNTIDYHK<br>TLAFDPKKGFWCQLLEGGKTKCLGKSKGRKYPEMDLDSRAFLKDYYRDHNIELSKLLYKMGQTL<br>PTWLREDLQNTR*
NDST (79–882) C-Avitag	ATGAAATTTTTGGTGAACGTGGCCTTGGTGTTTATGGTGGTTTACATTAGCTACATTTATGC<br>GGACCCGGGCCATCATCACCACCATCATGGTACCGCAGAAAACTTGTACTTTCAAGGCTCCC<br>GCACAGACCCGTTGGTGCTGGTCTTTGTGGAGAGCCTCTACTCGCAACTGGGCCAGGAGGTG<br>GTGGCCATCCTGGAGTCCAGCCGCTTCAAATACCGCACAGAGATTGCGCCGGGCAAGGGTGA<br>CATGCCCACGCTCACTGACAAGGGCCGTGGCCGCTTCGCCCTCATCATCTATGAGAACATCC<br>TCAAGTATGTCAACCTGGACGCCTGGAACCGGGAGCTGCTGGACAAGTACTGTGTGGCCTAC<br>GGCGTGGGCATCATTGGCTTCTTCAAGGCCAATGAGAACAGCCTGCTGAGTGCGCAGCTCAA<br>GGGCTTCCCCCTGTTCCTGCACTCAAACCTGGGCCTGAAGGACTGCAGCATCAACCCCAAGT<br>CCCCGCTGCTCTACGTGACGCGACCTAGCGAGGTGGAGAAAGGTGTGCTCCCCGGCGAGGAC<br>TGGACGGTTTTCCAGTCAAATCACTCCACCTATGAGCCAGTGCTGCTGGCCAAGACGCGCTC	MKFLVNVALVFMVVYISYIYADPGHHHHHHGTAENLYFQGSRTDPLVLVFVESLYSQLGQEVVA<br>ILESSRFKYRTEIAPGKGDMPTLTDKGRGRFALIIYENILKYVNLDAWNRELLDKYCVAYGVGI<br>IGFFKANENSLLSAQLKGFPLFLHSNLGLKDCSINPKSPLLYVTRPSEVEKGVLPGEDWTVFQS<br>NHSTYEPVLLAKTRSSESIPHLGADAGLHAALHATVVQDLGLHDGIQRVLFGNNLNFWLHKLVF<br>VDAVAFLTGKRLSLPLDRYILVDIDDIFVGKEGTRMKVEDVKALFDTQNELRAHIPNFTFNLGY<br>SGKFFHTGTNAEDAGDDLLLSYVKEFWWFPHMWSHMQPHLFHNQSVLAEQMALNKKFAVEHGIP<br>TDMGYAVAPHHSGVYPVHVQLYEAWKQVWSIRVTSTEEYPHLKPARYRRGFIHNGIMVLPRQTC<br>GLFTHTIFYNEYPGGSSELDKIINGGELFLTVLLNPISIFMTHLSNYGNDRLGLYTFKHLVRFL<br>HSWTNLRLQTLPPVQLAQKYFQIFSEEKDPLWQDPCEDKRHKDIWSKEKTCDRFPKLLIIGPQK<br>TGTTALYLFLGMHPDLSSNYPSSETFEEIQFFNGHNYHKGIDWYMEFFPIPSNTTSDFYFEKSA

	GTCAGAGTCCATCCCACACCTGGGCGCAGACGCCGGCCTGCATGCTGCACTGCACGCCACTG<br>TGGTCCAGGACCTGGGCCTGCACGACGGCATCCAGCGCGTGCTGTTTGGCAACAACCTGAAC<br>TTCTGGCTGCACAAGCTTGTCTTCGTGGATGCCGTGGCCTTCCTCACGGGGAAGCGCCTCTC<br>CCTGCCATTGGACCGCTACATCCTGGTGGACATTGATGACATCTTCGTGGGCAAGGAGGGCA<br>CACGCATGAAGGTGGAGGACGTGAAGGCCCTGTTTGACACACAGAACGAACTACGCGCACAC<br>ATCCCAAACTTCACCTTCAACCTGGGCTACTCAGGGAAATTCTTCCACACAGGTACCAATGC<br>TGAGGACGCTGGGGATGATCTGCTGCTGTCGTATGTGAAGGAGTTCTGGTGGTTCCCCCACA<br>TGTGGAGCCACATGCAGCCCCACCTTTTCCACAACCAGTCCGTGTTGGCCGAGCAGATGGCC<br>TTGAACAAGAAGTTCGCTGTCGAGCATGGCATTCCCACAGACATGGGGTATGCAGTGGCGCC<br>CCACCACTCGGGCGTGTACCCCGTGCACGTGCAGCTGTACGAGGCTTGGAAGCAAGTGTGGA<br>GCATCCGCGTGACCAGCACGGAGGAGTACCCCCACCTGAAGCCAGCCCGCTACCGCCGTGGC<br>TTCATCCACAATGGCATCATGGTTCTCCCACGGCAGACCTGCGGCCTCTTCACACACACCAT<br>CTTCTACAACGAGTACCCTGGCGGCTCCAGTGAGCTGGACAAGATCATCAACGGGGGCGAGC<br>TCTTCCTCACCGTGCTCCTCAATCCTATCAGCATCTTCATGACGCACCTGTCCAACTATGGG<br>AATGACCGCCTGGGCCTGTACACCTTCAAGCACCTGGTGCGCTTCCTGCACTCCTGGACGAA<br>CCTCCGGCTGCAGACACTGCCCCCTGTGCAGTTGGCGCAGAAGTACTTCCAGATCTTCTCCG<br>AGGAGAAGGACCCGCTCTGGCAGGACCCCTGCGAGGACAAACGTCACAAAGACATCTGGTCC<br>AAGGAGAAGACGTGTGACCGCTTCCCAAAGCTCCTCATCATCGGCCCCCAGAAAACAGGCAC<br>CACTGCCCTCTACCTGTTCCTGGGCATGCACCCTGACCTAAGCAGCAACTACCCCAGCTCTG<br>AGACATTTGAGGAGATCCAGTTTTTTAATGGCCACAACTATCACAAAGGCATCGACTGGTAC<br>ATGGAGTTCTTCCCCATCCCTTCCAACACCACGTCCGACTTCTACTTTGAGAAAAGCGCCAA<br>CTACTTTGATTCAGAAGTGGCGCCCCGGCGGGCAGCAGCCCTCTTGCCCAAAGCCAAGGTCC<br>TGACCATCCTCATCAACCCCGCGGACCGGGCCTATTCCTGGTACCAGCACCAGCGAGCCCAT<br>GACGACCCAGTGGCCCTAAAGTACACCTTCCATGAGGTGATTACCGCCGGCTCTGACGCATC<br>CTCGAAGCTGCGTGCCCTCCAGAACCGCTGCCTGGTCCCTGGCTGGTACGCCACCCACATCG<br>AGCGCTGGCTCAGTGCCTATCACGCCAACCAGATTCTGGTCTTGGATGGCAAACTGCTTCGC<br>ACAGAACCTGCCAAAGTGATGGACATGGTGCAGAAGTTCCTTGGGGTGACCAACACCATTGA<br>CTACCACAAAACCTTGGCGTTTGATCCAAAGAAAGGATTTTGGTGCCAACTGCTTGAAGGAG<br>GAAAAACCAAGTGTCTGGGCAAAAGCAAGGGCCGGAAATATCCCGAGATGGACTTGGATTCC<br>CGAGCCTTCCTGAAGGACTATTACCGGGACCACAACATCGAGCTCTCCAAGCTGCTGTATAA<br>GATGGGCCAGACACTTCCCACTTGGCTACGAGAGGACCTCCAGAACACCAGGTCAAGCGGAG<br>GCCTGAACGACATCTTCGAGGCCCAGAAAATCGAGTGGCACGAGTAA	NYFDSEVAPRRAAALLPKAKVLTILINPADRAYSWYQHQRAHDDPVALKYTFHEVITAGSDASS<br>KLRALQNRCLVPGWYATHIERWLSAYHANQILVLDGKLLRTEPAKVMDMVQKFLGVTNTIDYHK<br>TLAFDPKKGFWCQLLEGGKTKCLGKSKGRKYPEMDLDSRAFLKDYYRDHNIELSKLLYKMGQTL<br>PTWLREDLQNTRSSGGLNDIFEAQKIEWHE*
NDST (79–882) – D319A	ATGAAATTTTTGGTGAACGTGGCCTTGGTGTTTATGGTGGTTTACATTAGCTACATTTATGC<br>GGACCCGGGCCATCATCACCACCATCATGGTACCGCAGAAAACTTGTACTTTCAAGGCTCCC<br>GCACAGACCCGTTGGTGCTGGTCTTTGTGGAGAGCCTCTACTCGCAACTGGGCCAGGAGGTG<br>GTGGCCATCCTGGAGTCCAGCCGCTTCAAATACCGCACAGAGATTGCGCCGGGCAAGGGTGA<br>CATGCCCACGCTCACTGACAAGGGCCGTGGCCGCTTCGCCCTCATCATCTATGAGAACATCC<br>TCAAGTATGTCAACCTGGACGCCTGGAACCGGGAGCTGCTGGACAAGTACTGTGTGGCCTAC<br>GGCGTGGGCATCATTGGCTTCTTCAAGGCCAATGAGAACAGCCTGCTGAGTGCGCAGCTCAA<br>GGGCTTCCCCCTGTTCCTGCACTCAAACCTGGGCCTGAAGGACTGCAGCATCAACCCCAAGT<br>CCCCGCTGCTCTACGTGACGCGACCTAGCGAGGTGGAGAAAGGTGTGCTCCCCGGCGAGGAC<br>TGGACGGTTTTCCAGTCAAATCACTCCACCTATGAGCCAGTGCTGCTGGCCAAGACGCGCTC<br>GTCAGAGTCCATCCCACACCTGGGCGCAGACGCCGGCCTGCATGCTGCACTGCACGCCACTG<br>TGGTCCAGGACCTGGGCCTGCACGACGGCATCCAGCGCGTGCTGTTTGGCAACAACCTGAAC<br>TTCTGGCTGCACAAGCTTGTCTTCGTGGATGCCGTGGCCTTCCTCACGGGGAAGCGCCTCTC<br>CCTGCCATTGGACCGCTACATCCTGGTGGACATTGCGGACATCTTCGTGGGCAAGGAGGGCA<br>CACGCATGAAGGTGGAGGACGTGAAGGCCCTGTTTGACACACAGAACGAACTACGCGCACAC<br>ATCCCAAACTTCACCTTCAACCTGGGCTACTCAGGGAAATTCTTCCACACAGGTACCAATGC<br>TGAGGACGCTGGGGATGATCTGCTGCTGTCGTATGTGAAGGAGTTCTGGTGGTTCCCCCACA<br>TGTGGAGCCACATGCAGCCCCACCTTTTCCACAACCAGTCCGTGTTGGCCGAGCAGATGGCC<br>TTGAACAAGAAGTTCGCTGTCGAGCATGGCATTCCCACAGACATGGGGTATGCAGTGGCGCC<br>CCACCACTCGGGCGTGTACCCCGTGCACGTGCAGCTGTACGAGGCTTGGAAGCAAGTGTGGA<br>GCATCCGCGTGACCAGCACGGAGGAGTACCCCCACCTGAAGCCAGCCCGCTACCGCCGTGGC<br>TTCATCCACAATGGCATCATGGTTCTCCCACGGCAGACCTGCGGCCTCTTCACACACACCAT<br>CTTCTACAACGAGTACCCTGGCGGCTCCAGTGAGCTGGACAAGATCATCAACGGGGGCGAGC<br>TCTTCCTCACCGTGCTCCTCAATCCTATCAGCATCTTCATGACGCACCTGTCCAACTATGGG	MKFLVNVALVFMVVYISYIYADPGHHHHHHGTAENLYFQGSRTDPLVLVFVESLYSQLGQEVVA<br>ILESSRFKYRTEIAPGKGDMPTLTDKGRGRFALIIYENILKYVNLDAWNRELLDKYCVAYGVGI<br>IGFFKANENSLLSAQLKGFPLFLHSNLGLKDCSINPKSPLLYVTRPSEVEKGVLPGEDWTVFQS<br>NHSTYEPVLLAKTRSSESIPHLGADAGLHAALHATVVQDLGLHDGIQRVLFGNNLNFWLHKLVF<br>VDAVAFLTGKRLSLPLDRYILVDIADIFVGKEGTRMKVEDVKALFDTQNELRAHIPNFTFNLGY<br>SGKFFHTGTNAEDAGDDLLLSYVKEFWWFPHMWSHMQPHLFHNQSVLAEQMALNKKFAVEHGIP<br>TDMGYAVAPHHSGVYPVHVQLYEAWKQVWSIRVTSTEEYPHLKPARYRRGFIHNGIMVLPRQTC<br>GLFTHTIFYNEYPGGSSELDKIINGGELFLTVLLNPISIFMTHLSNYGNDRLGLYTFKHLVRFL<br>HSWTNLRLQTLPPVQLAQKYFQIFSEEKDPLWQDPCEDKRHKDIWSKEKTCDRFPKLLIIGPQK<br>TGTTALYLFLGMHPDLSSNYPSSETFEEIQFFNGHNYHKGIDWYMEFFPIPSNTTSDFYFEKSA<br>NYFDSEVAPRRAAALLPKAKVLTILINPADRAYSWYQHQRAHDDPVALKYTFHEVITAGSDASS<br>KLRALQNRCLVPGWYATHIERWLSAYHANQILVLDGKLLRTEPAKVMDMVQKFLGVTNTIDYHK<br>TLAFDPKKGFWCQLLEGGKTKCLGKSKGRKYPEMDLDSRAFLKDYYRDHNIELSKLLYKMGQTL<br>PTWLREDLQNTR*

	AATGACCGCCTGGGCCTGTACACCTTCAAGCACCTGGTGCGCTTCCTGCACTCCTGGACGAA<br>CCTCCGGCTGCAGACACTGCCCCCTGTGCAGTTGGCGCAGAAGTACTTCCAGATCTTCTCCG<br>AGGAGAAGGACCCGCTCTGGCAGGACCCCTGCGAGGACAAACGTCACAAAGACATCTGGTCC<br>AAGGAGAAGACGTGTGACCGCTTCCCAAAGCTCCTCATCATCGGCCCCCAGAAAACAGGCAC<br>CACTGCCCTCTACCTGTTCCTGGGCATGCACCCTGACCTAAGCAGCAACTACCCCAGCTCTG<br>AGACATTTGAGGAGATCCAGTTTTTTAATGGCCACAACTATCACAAAGGCATCGACTGGTAC<br>ATGGAGTTCTTCCCCATCCCTTCCAACACCACGTCCGACTTCTACTTTGAGAAAAGCGCCAA<br>CTACTTTGATTCAGAAGTGGCGCCCCGGCGGGCAGCAGCCCTCTTGCCCAAAGCCAAGGTCC<br>TGACCATCCTCATCAACCCCGCGGACCGGGCCTATTCCTGGTACCAGCACCAGCGAGCCCAT<br>GACGACCCAGTGGCCCTAAAGTACACCTTCCATGAGGTGATTACCGCCGGCTCTGACGCATC<br>CTCGAAGCTGCGTGCCCTCCAGAACCGCTGCCTGGTCCCTGGCTGGTACGCCACCCACATCG<br>AGCGCTGGCTCAGTGCCTATCACGCCAACCAGATTCTGGTCTTGGATGGCAAACTGCTTCGC<br>ACAGAACCTGCCAAAGTGATGGACATGGTGCAGAAGTTCCTTGGGGTGACCAACACCATTGA<br>CTACCACAAAACCTTGGCGTTTGATCCAAAGAAAGGATTTTGGTGCCAACTGCTTGAAGGAG<br>GAAAAACCAAGTGTCTGGGCAAAAGCAAGGGCCGGAAATATCCCGAGATGGACTTGGATTCC<br>CGAGCCTTCCTGAAGGACTATTACCGGGACCACAACATCGAGCTCTCCAAGCTGCTGTATAA<br>GATGGGCCAGACACTTCCCACTTGGCTACGAGAGGACCTCCAGAACACCAGGTAA	
NDST (79–882) – D320A	ATGAAATTTTTGGTGAACGTGGCCTTGGTGTTTATGGTGGTTTACATTAGCTACATTTATGC<br>GGACCCGGGCCATCATCACCACCATCATGGTACCGCAGAAAACTTGTACTTTCAAGGCTCCC<br>GCACAGACCCGTTGGTGCTGGTCTTTGTGGAGAGCCTCTACTCGCAACTGGGCCAGGAGGTG<br>GTGGCCATCCTGGAGTCCAGCCGCTTCAAATACCGCACAGAGATTGCGCCGGGCAAGGGTGA<br>CATGCCCACGCTCACTGACAAGGGCCGTGGCCGCTTCGCCCTCATCATCTATGAGAACATCC<br>TCAAGTATGTCAACCTGGACGCCTGGAACCGGGAGCTGCTGGACAAGTACTGTGTGGCCTAC<br>GGCGTGGGCATCATTGGCTTCTTCAAGGCCAATGAGAACAGCCTGCTGAGTGCGCAGCTCAA<br>GGGCTTCCCCCTGTTCCTGCACTCAAACCTGGGCCTGAAGGACTGCAGCATCAACCCCAAGT<br>CCCCGCTGCTCTACGTGACGCGACCTAGCGAGGTGGAGAAAGGTGTGCTCCCCGGCGAGGAC<br>TGGACGGTTTTCCAGTCAAATCACTCCACCTATGAGCCAGTGCTGCTGGCCAAGACGCGCTC<br>GTCAGAGTCCATCCCACACCTGGGCGCAGACGCCGGCCTGCATGCTGCACTGCACGCCACTG<br>TGGTCCAGGACCTGGGCCTGCACGACGGCATCCAGCGCGTGCTGTTTGGCAACAACCTGAAC<br>TTCTGGCTGCACAAGCTTGTCTTCGTGGATGCCGTGGCCTTCCTCACGGGGAAGCGCCTCTC<br>CCTGCCATTGGACCGCTACATCCTGGTGGACATTGATGCGATCTTCGTGGGCAAGGAGGGCA<br>CACGCATGAAGGTGGAGGACGTGAAGGCCCTGTTTGACACACAGAACGAACTACGCGCACAC<br>ATCCCAAACTTCACCTTCAACCTGGGCTACTCAGGGAAATTCTTCCACACAGGTACCAATGC<br>TGAGGACGCTGGGGATGATCTGCTGCTGTCGTATGTGAAGGAGTTCTGGTGGTTCCCCCACA<br>TGTGGAGCCACATGCAGCCCCACCTTTTCCACAACCAGTCCGTGTTGGCCGAGCAGATGGCC<br>TTGAACAAGAAGTTCGCTGTCGAGCATGGCATTCCCACAGACATGGGGTATGCAGTGGCGCC<br>CCACCACTCGGGCGTGTACCCCGTGCACGTGCAGCTGTACGAGGCTTGGAAGCAAGTGTGGA<br>GCATCCGCGTGACCAGCACGGAGGAGTACCCCCACCTGAAGCCAGCCCGCTACCGCCGTGGC<br>TTCATCCACAATGGCATCATGGTTCTCCCACGGCAGACCTGCGGCCTCTTCACACACACCAT<br>CTTCTACAACGAGTACCCTGGCGGCTCCAGTGAGCTGGACAAGATCATCAACGGGGGCGAGC<br>TCTTCCTCACCGTGCTCCTCAATCCTATCAGCATCTTCATGACGCACCTGTCCAACTATGGG<br>AATGACCGCCTGGGCCTGTACACCTTCAAGCACCTGGTGCGCTTCCTGCACTCCTGGACGAA<br>CCTCCGGCTGCAGACACTGCCCCCTGTGCAGTTGGCGCAGAAGTACTTCCAGATCTTCTCCG<br>AGGAGAAGGACCCGCTCTGGCAGGACCCCTGCGAGGACAAACGTCACAAAGACATCTGGTCC<br>AAGGAGAAGACGTGTGACCGCTTCCCAAAGCTCCTCATCATCGGCCCCCAGAAAACAGGCAC<br>CACTGCCCTCTACCTGTTCCTGGGCATGCACCCTGACCTAAGCAGCAACTACCCCAGCTCTG<br>AGACATTTGAGGAGATCCAGTTTTTTAATGGCCACAACTATCACAAAGGCATCGACTGGTAC<br>ATGGAGTTCTTCCCCATCCCTTCCAACACCACGTCCGACTTCTACTTTGAGAAAAGCGCCAA<br>CTACTTTGATTCAGAAGTGGCGCCCCGGCGGGCAGCAGCCCTCTTGCCCAAAGCCAAGGTCC<br>TGACCATCCTCATCAACCCCGCGGACCGGGCCTATTCCTGGTACCAGCACCAGCGAGCCCAT<br>GACGACCCAGTGGCCCTAAAGTACACCTTCCATGAGGTGATTACCGCCGGCTCTGACGCATC<br>CTCGAAGCTGCGTGCCCTCCAGAACCGCTGCCTGGTCCCTGGCTGGTACGCCACCCACATCG<br>AGCGCTGGCTCAGTGCCTATCACGCCAACCAGATTCTGGTCTTGGATGGCAAACTGCTTCGC<br>ACAGAACCTGCCAAAGTGATGGACATGGTGCAGAAGTTCCTTGGGGTGACCAACACCATTGA<br>CTACCACAAAACCTTGGCGTTTGATCCAAAGAAAGGATTTTGGTGCCAACTGCTTGAAGGAG<br>GAAAAACCAAGTGTCTGGGCAAAAGCAAGGGCCGGAAATATCCCGAGATGGACTTGGATTCC	MKFLVNVALVFMVVYISYIYADPGHHHHHHGTAENLYFQGSRTDPLVLVFVESLYSQLGQEVVA<br>ILESSRFKYRTEIAPGKGDMPTLTDKGRGRFALIIYENILKYVNLDAWNRELLDKYCVAYGVGI<br>IGFFKANENSLLSAQLKGFPLFLHSNLGLKDCSINPKSPLLYVTRPSEVEKGVLPGEDWTVFQS<br>NHSTYEPVLLAKTRSSESIPHLGADAGLHAALHATVVQDLGLHDGIQRVLFGNNLNFWLHKLVF<br>VDAVAFLTGKRLSLPLDRYILVDIDAIFVGKEGTRMKVEDVKALFDTQNELRAHIPNFTFNLGY<br>SGKFFHTGTNAEDAGDDLLLSYVKEFWWFPHMWSHMQPHLFHNQSVLAEQMALNKKFAVEHGIP<br>TDMGYAVAPHHSGVYPVHVQLYEAWKQVWSIRVTSTEEYPHLKPARYRRGFIHNGIMVLPRQTC<br>GLFTHTIFYNEYPGGSSELDKIINGGELFLTVLLNPISIFMTHLSNYGNDRLGLYTFKHLVRFL<br>HSWTNLRLQTLPPVQLAQKYFQIFSEEKDPLWQDPCEDKRHKDIWSKEKTCDRFPKLLIIGPQK<br>TGTTALYLFLGMHPDLSSNYPSSETFEEIQFFNGHNYHKGIDWYMEFFPIPSNTTSDFYFEKSA<br>NYFDSEVAPRRAAALLPKAKVLTILINPADRAYSWYQHQRAHDDPVALKYTFHEVITAGSDASS<br>KLRALQNRCLVPGWYATHIERWLSAYHANQILVLDGKLLRTEPAKVMDMVQKFLGVTNTIDYHK<br>TLAFDPKKGFWCQLLEGGKTKCLGKSKGRKYPEMDLDSRAFLKDYYRDHNIELSKLLYKMGQTL<br>PTWLREDLQNTR*

	CGAGCCTTCCTGAAGGACTATTACCGGGACCACAACATCGAGCTCTCCAAGCTGCTGTATAA<br>GATGGGCCAGACACTTCCCACTTGGCTACGAGAGGACCTCCAGAACACCAGGTAA	
NDST (79–882) – H389A	ATGAAATTTTTGGTGAACGTGGCCTTGGTGTTTATGGTGGTTTACATTAGCTACATTTATGC<br>GGACCCGGGCCATCATCACCACCATCATGGTACCGCAGAAAACTTGTACTTTCAAGGCTCCC<br>GCACAGACCCGTTGGTGCTGGTCTTTGTGGAGAGCCTCTACTCGCAACTGGGCCAGGAGGTG<br>GTGGCCATCCTGGAGTCCAGCCGCTTCAAATACCGCACAGAGATTGCGCCGGGCAAGGGTGA<br>CATGCCCACGCTCACTGACAAGGGCCGTGGCCGCTTCGCCCTCATCATCTATGAGAACATCC<br>TCAAGTATGTCAACCTGGACGCCTGGAACCGGGAGCTGCTGGACAAGTACTGTGTGGCCTAC<br>GGCGTGGGCATCATTGGCTTCTTCAAGGCCAATGAGAACAGCCTGCTGAGTGCGCAGCTCAA<br>GGGCTTCCCCCTGTTCCTGCACTCAAACCTGGGCCTGAAGGACTGCAGCATCAACCCCAAGT<br>CCCCGCTGCTCTACGTGACGCGACCTAGCGAGGTGGAGAAAGGTGTGCTCCCCGGCGAGGAC<br>TGGACGGTTTTCCAGTCAAATCACTCCACCTATGAGCCAGTGCTGCTGGCCAAGACGCGCTC<br>GTCAGAGTCCATCCCACACCTGGGCGCAGACGCCGGCCTGCATGCTGCACTGCACGCCACTG<br>TGGTCCAGGACCTGGGCCTGCACGACGGCATCCAGCGCGTGCTGTTTGGCAACAACCTGAAC<br>TTCTGGCTGCACAAGCTTGTCTTCGTGGATGCCGTGGCCTTCCTCACGGGGAAGCGCCTCTC<br>CCTGCCATTGGACCGCTACATCCTGGTGGACATTGATGACATCTTCGTGGGCAAGGAGGGCA<br>CACGCATGAAGGTGGAGGACGTGAAGGCCCTGTTTGACACACAGAACGAACTACGCGCACAC<br>ATCCCAAACTTCACCTTCAACCTGGGCTACTCAGGGAAATTCTTCCACACAGGTACCAATGC<br>TGAGGACGCTGGGGATGATCTGCTGCTGTCGTATGTGAAGGAGTTCTGGTGGTTCCCCGCGA<br>TGTGGAGCCACATGCAGCCCCACCTTTTCCACAACCAGTCCGTGTTGGCCGAGCAGATGGCC<br>TTGAACAAGAAGTTCGCTGTCGAGCATGGCATTCCCACAGACATGGGGTATGCAGTGGCGCC<br>CCACCACTCGGGCGTGTACCCCGTGCACGTGCAGCTGTACGAGGCTTGGAAGCAAGTGTGGA<br>GCATCCGCGTGACCAGCACGGAGGAGTACCCCCACCTGAAGCCAGCCCGCTACCGCCGTGGC<br>TTCATCCACAATGGCATCATGGTTCTCCCACGGCAGACCTGCGGCCTCTTCACACACACCAT<br>CTTCTACAACGAGTACCCTGGCGGCTCCAGTGAGCTGGACAAGATCATCAACGGGGGCGAGC<br>TCTTCCTCACCGTGCTCCTCAATCCTATCAGCATCTTCATGACGCACCTGTCCAACTATGGG<br>AATGACCGCCTGGGCCTGTACACCTTCAAGCACCTGGTGCGCTTCCTGCACTCCTGGACGAA<br>CCTCCGGCTGCAGACACTGCCCCCTGTGCAGTTGGCGCAGAAGTACTTCCAGATCTTCTCCG<br>AGGAGAAGGACCCGCTCTGGCAGGACCCCTGCGAGGACAAACGTCACAAAGACATCTGGTCC<br>AAGGAGAAGACGTGTGACCGCTTCCCAAAGCTCCTCATCATCGGCCCCCAGAAAACAGGCAC<br>CACTGCCCTCTACCTGTTCCTGGGCATGCACCCTGACCTAAGCAGCAACTACCCCAGCTCTG<br>AGACATTTGAGGAGATCCAGTTTTTTAATGGCCACAACTATCACAAAGGCATCGACTGGTAC<br>ATGGAGTTCTTCCCCATCCCTTCCAACACCACGTCCGACTTCTACTTTGAGAAAAGCGCCAA<br>CTACTTTGATTCAGAAGTGGCGCCCCGGCGGGCAGCAGCCCTCTTGCCCAAAGCCAAGGTCC<br>TGACCATCCTCATCAACCCCGCGGACCGGGCCTATTCCTGGTACCAGCACCAGCGAGCCCAT<br>GACGACCCAGTGGCCCTAAAGTACACCTTCCATGAGGTGATTACCGCCGGCTCTGACGCATC<br>CTCGAAGCTGCGTGCCCTCCAGAACCGCTGCCTGGTCCCTGGCTGGTACGCCACCCACATCG<br>AGCGCTGGCTCAGTGCCTATCACGCCAACCAGATTCTGGTCTTGGATGGCAAACTGCTTCGC<br>ACAGAACCTGCCAAAGTGATGGACATGGTGCAGAAGTTCCTTGGGGTGACCAACACCATTGA<br>CTACCACAAAACCTTGGCGTTTGATCCAAAGAAAGGATTTTGGTGCCAACTGCTTGAAGGAG<br>GAAAAACCAAGTGTCTGGGCAAAAGCAAGGGCCGGAAATATCCCGAGATGGACTTGGATTCC<br>CGAGCCTTCCTGAAGGACTATTACCGGGACCACAACATCGAGCTCTCCAAGCTGCTGTATAA<br>GATGGGCCAGACACTTCCCACTTGGCTACGAGAGGACCTCCAGAACACCAGGTAA	MKFLVNVALVFMVVYISYIYADPGHHHHHHGTAENLYFQGSRTDPLVLVFVESLYSQLGQEVVA<br>ILESSRFKYRTEIAPGKGDMPTLTDKGRGRFALIIYENILKYVNLDAWNRELLDKYCVAYGVGI<br>IGFFKANENSLLSAQLKGFPLFLHSNLGLKDCSINPKSPLLYVTRPSEVEKGVLPGEDWTVFQS<br>NHSTYEPVLLAKTRSSESIPHLGADAGLHAALHATVVQDLGLHDGIQRVLFGNNLNFWLHKLVF<br>VDAVAFLTGKRLSLPLDRYILVDIDDIFVGKEGTRMKVEDVKALFDTQNELRAHIPNFTFNLGY<br>SGKFFHTGTNAEDAGDDLLLSYVKEFWWFPAMWSHMQPHLFHNQSVLAEQMALNKKFAVEHGIP<br>TDMGYAVAPHHSGVYPVHVQLYEAWKQVWSIRVTSTEEYPHLKPARYRRGFIHNGIMVLPRQTC<br>GLFTHTIFYNEYPGGSSELDKIINGGELFLTVLLNPISIFMTHLSNYGNDRLGLYTFKHLVRFL<br>HSWTNLRLQTLPPVQLAQKYFQIFSEEKDPLWQDPCEDKRHKDIWSKEKTCDRFPKLLIIGPQK<br>TGTTALYLFLGMHPDLSSNYPSSETFEEIQFFNGHNYHKGIDWYMEFFPIPSNTTSDFYFEKSA<br>NYFDSEVAPRRAAALLPKAKVLTILINPADRAYSWYQHQRAHDDPVALKYTFHEVITAGSDASS<br>KLRALQNRCLVPGWYATHIERWLSAYHANQILVLDGKLLRTEPAKVMDMVQKFLGVTNTIDYHK<br>TLAFDPKKGFWCQLLEGGKTKCLGKSKGRKYPEMDLDSRAFLKDYYRDHNIELSKLLYKMGQTL<br>PTWLREDLQNTR*
NDST (79–882) – H393A	ATGAAATTTTTGGTGAACGTGGCCTTGGTGTTTATGGTGGTTTACATTAGCTACATTTATGC<br>GGACCCGGGCCATCATCACCACCATCATGGTACCGCAGAAAACTTGTACTTTCAAGGCTCCC<br>GCACAGACCCGTTGGTGCTGGTCTTTGTGGAGAGCCTCTACTCGCAACTGGGCCAGGAGGTG<br>GTGGCCATCCTGGAGTCCAGCCGCTTCAAATACCGCACAGAGATTGCGCCGGGCAAGGGTGA<br>CATGCCCACGCTCACTGACAAGGGCCGTGGCCGCTTCGCCCTCATCATCTATGAGAACATCC<br>TCAAGTATGTCAACCTGGACGCCTGGAACCGGGAGCTGCTGGACAAGTACTGTGTGGCCTAC<br>GGCGTGGGCATCATTGGCTTCTTCAAGGCCAATGAGAACAGCCTGCTGAGTGCGCAGCTCAA<br>GGGCTTCCCCCTGTTCCTGCACTCAAACCTGGGCCTGAAGGACTGCAGCATCAACCCCAAGT<br>CCCCGCTGCTCTACGTGACGCGACCTAGCGAGGTGGAGAAAGGTGTGCTCCCCGGCGAGGAC<br>TGGACGGTTTTCCAGTCAAATCACTCCACCTATGAGCCAGTGCTGCTGGCCAAGACGCGCTC<br>GTCAGAGTCCATCCCACACCTGGGCGCAGACGCCGGCCTGCATGCTGCACTGCACGCCACTG<br>TGGTCCAGGACCTGGGCCTGCACGACGGCATCCAGCGCGTGCTGTTTGGCAACAACCTGAAC<br>TTCTGGCTGCACAAGCTTGTCTTCGTGGATGCCGTGGCCTTCCTCACGGGGAAGCGCCTCTC	MKFLVNVALVFMVVYISYIYADPGHHHHHHGTAENLYFQGSRTDPLVLVFVESLYSQLGQEVVA<br>ILESSRFKYRTEIAPGKGDMPTLTDKGRGRFALIIYENILKYVNLDAWNRELLDKYCVAYGVGI<br>IGFFKANENSLLSAQLKGFPLFLHSNLGLKDCSINPKSPLLYVTRPSEVEKGVLPGEDWTVFQS<br>NHSTYEPVLLAKTRSSESIPHLGADAGLHAALHATVVQDLGLHDGIQRVLFGNNLNFWLHKLVF<br>VDAVAFLTGKRLSLPLDRYILVDIDDIFVGKEGTRMKVEDVKALFDTQNELRAHIPNFTFNLGY<br>SGKFFHTGTNAEDAGDDLLLSYVKEFWWFPHMWSAMQPHLFHNQSVLAEQMALNKKFAVEHGIP<br>TDMGYAVAPHHSGVYPVHVQLYEAWKQVWSIRVTSTEEYPHLKPARYRRGFIHNGIMVLPRQTC<br>GLFTHTIFYNEYPGGSSELDKIINGGELFLTVLLNPISIFMTHLSNYGNDRLGLYTFKHLVRFL<br>HSWTNLRLQTLPPVQLAQKYFQIFSEEKDPLWQDPCEDKRHKDIWSKEKTCDRFPKLLIIGPQK<br>TGTTALYLFLGMHPDLSSNYPSSETFEEIQFFNGHNYHKGIDWYMEFFPIPSNTTSDFYFEKSA<br>NYFDSEVAPRRAAALLPKAKVLTILINPADRAYSWYQHQRAHDDPVALKYTFHEVITAGSDASS<br>KLRALQNRCLVPGWYATHIERWLSAYHANQILVLDGKLLRTEPAKVMDMVQKFLGVTNTIDYHK

	CCTGCCATTGGACCGCTACATCCTGGTGGACATTGATGACATCTTCGTGGGCAAGGAGGGCA<br>CACGCATGAAGGTGGAGGACGTGAAGGCCCTGTTTGACACACAGAACGAACTACGCGCACAC<br>ATCCCAAACTTCACCTTCAACCTGGGCTACTCAGGGAAATTCTTCCACACAGGTACCAATGC<br>TGAGGACGCTGGGGATGATCTGCTGCTGTCGTATGTGAAGGAGTTCTGGTGGTTCCCCCACA<br>TGTGGAGCGCGATGCAGCCCCACCTTTTCCACAACCAGTCCGTGTTGGCCGAGCAGATGGCC<br>TTGAACAAGAAGTTCGCTGTCGAGCATGGCATTCCCACAGACATGGGGTATGCAGTGGCGCC<br>CCACCACTCGGGCGTGTACCCCGTGCACGTGCAGCTGTACGAGGCTTGGAAGCAAGTGTGGA<br>GCATCCGCGTGACCAGCACGGAGGAGTACCCCCACCTGAAGCCAGCCCGCTACCGCCGTGGC<br>TTCATCCACAATGGCATCATGGTTCTCCCACGGCAGACCTGCGGCCTCTTCACACACACCAT<br>CTTCTACAACGAGTACCCTGGCGGCTCCAGTGAGCTGGACAAGATCATCAACGGGGGCGAGC<br>TCTTCCTCACCGTGCTCCTCAATCCTATCAGCATCTTCATGACGCACCTGTCCAACTATGGG<br>AATGACCGCCTGGGCCTGTACACCTTCAAGCACCTGGTGCGCTTCCTGCACTCCTGGACGAA<br>CCTCCGGCTGCAGACACTGCCCCCTGTGCAGTTGGCGCAGAAGTACTTCCAGATCTTCTCCG<br>AGGAGAAGGACCCGCTCTGGCAGGACCCCTGCGAGGACAAACGTCACAAAGACATCTGGTCC<br>AAGGAGAAGACGTGTGACCGCTTCCCAAAGCTCCTCATCATCGGCCCCCAGAAAACAGGCAC<br>CACTGCCCTCTACCTGTTCCTGGGCATGCACCCTGACCTAAGCAGCAACTACCCCAGCTCTG<br>AGACATTTGAGGAGATCCAGTTTTTTAATGGCCACAACTATCACAAAGGCATCGACTGGTAC<br>ATGGAGTTCTTCCCCATCCCTTCCAACACCACGTCCGACTTCTACTTTGAGAAAAGCGCCAA<br>CTACTTTGATTCAGAAGTGGCGCCCCGGCGGGCAGCAGCCCTCTTGCCCAAAGCCAAGGTCC<br>TGACCATCCTCATCAACCCCGCGGACCGGGCCTATTCCTGGTACCAGCACCAGCGAGCCCAT<br>GACGACCCAGTGGCCCTAAAGTACACCTTCCATGAGGTGATTACCGCCGGCTCTGACGCATC<br>CTCGAAGCTGCGTGCCCTCCAGAACCGCTGCCTGGTCCCTGGCTGGTACGCCACCCACATCG<br>AGCGCTGGCTCAGTGCCTATCACGCCAACCAGATTCTGGTCTTGGATGGCAAACTGCTTCGC<br>ACAGAACCTGCCAAAGTGATGGACATGGTGCAGAAGTTCCTTGGGGTGACCAACACCATTGA<br>CTACCACAAAACCTTGGCGTTTGATCCAAAGAAAGGATTTTGGTGCCAACTGCTTGAAGGAG<br>GAAAAACCAAGTGTCTGGGCAAAAGCAAGGGCCGGAAATATCCCGAGATGGACTTGGATTCC<br>CGAGCCTTCCTGAAGGACTATTACCGGGACCACAACATCGAGCTCTCCAAGCTGCTGTATAA<br>GATGGGCCAGACACTTCCCACTTGGCTACGAGAGGACCTCCAGAACACCAGGTAA	TLAFDPKKGFWCQLLEGGKTKCLGKSKGRKYPEMDLDSRAFLKDYYRDHNIELSKLLYKMGQTL<br>PTWLREDLQNTR*
NDST (79–882) – H529A	ATGAAATTTTTGGTGAACGTGGCCTTGGTGTTTATGGTGGTTTACATTAGCTACATTTATGC<br>GGACCCGGGCCATCATCACCACCATCATGGTACCGCAGAAAACTTGTACTTTCAAGGCTCCC<br>GCACAGACCCGTTGGTGCTGGTCTTTGTGGAGAGCCTCTACTCGCAACTGGGCCAGGAGGTG<br>GTGGCCATCCTGGAGTCCAGCCGCTTCAAATACCGCACAGAGATTGCGCCGGGCAAGGGTGA<br>CATGCCCACGCTCACTGACAAGGGCCGTGGCCGCTTCGCCCTCATCATCTATGAGAACATCC<br>TCAAGTATGTCAACCTGGACGCCTGGAACCGGGAGCTGCTGGACAAGTACTGTGTGGCCTAC<br>GGCGTGGGCATCATTGGCTTCTTCAAGGCCAATGAGAACAGCCTGCTGAGTGCGCAGCTCAA<br>GGGCTTCCCCCTGTTCCTGCACTCAAACCTGGGCCTGAAGGACTGCAGCATCAACCCCAAGT<br>CCCCGCTGCTCTACGTGACGCGACCTAGCGAGGTGGAGAAAGGTGTGCTCCCCGGCGAGGAC<br>TGGACGGTTTTCCAGTCAAATCACTCCACCTATGAGCCAGTGCTGCTGGCCAAGACGCGCTC<br>GTCAGAGTCCATCCCACACCTGGGCGCAGACGCCGGCCTGCATGCTGCACTGCACGCCACTG<br>TGGTCCAGGACCTGGGCCTGCACGACGGCATCCAGCGCGTGCTGTTTGGCAACAACCTGAAC<br>TTCTGGCTGCACAAGCTTGTCTTCGTGGATGCCGTGGCCTTCCTCACGGGGAAGCGCCTCTC<br>CCTGCCATTGGACCGCTACATCCTGGTGGACATTGATGACATCTTCGTGGGCAAGGAGGGCA<br>CACGCATGAAGGTGGAGGACGTGAAGGCCCTGTTTGACACACAGAACGAACTACGCGCACAC<br>ATCCCAAACTTCACCTTCAACCTGGGCTACTCAGGGAAATTCTTCCACACAGGTACCAATGC<br>TGAGGACGCTGGGGATGATCTGCTGCTGTCGTATGTGAAGGAGTTCTGGTGGTTCCCCCACA<br>TGTGGAGCCACATGCAGCCCCACCTTTTCCACAACCAGTCCGTGTTGGCCGAGCAGATGGCC<br>TTGAACAAGAAGTTCGCTGTCGAGCATGGCATTCCCACAGACATGGGGTATGCAGTGGCGCC<br>CCACCACTCGGGCGTGTACCCCGTGCACGTGCAGCTGTACGAGGCTTGGAAGCAAGTGTGGA<br>GCATCCGCGTGACCAGCACGGAGGAGTACCCCCACCTGAAGCCAGCCCGCTACCGCCGTGGC<br>TTCATCCACAATGGCATCATGGTTCTCCCACGGCAGACCTGCGGCCTCTTCACACACACCAT<br>CTTCTACAACGAGTACCCTGGCGGCTCCAGTGAGCTGGACAAGATCATCAACGGGGGCGAGC<br>TCTTCCTCACCGTGCTCCTCAATCCTATCAGCATCTTCATGACGGCGCTGTCCAACTATGGG<br>AATGACCGCCTGGGCCTGTACACCTTCAAGCACCTGGTGCGCTTCCTGCACTCCTGGACGAA<br>CCTCCGGCTGCAGACACTGCCCCCTGTGCAGTTGGCGCAGAAGTACTTCCAGATCTTCTCCG<br>AGGAGAAGGACCCGCTCTGGCAGGACCCCTGCGAGGACAAACGTCACAAAGACATCTGGTCC<br>AAGGAGAAGACGTGTGACCGCTTCCCAAAGCTCCTCATCATCGGCCCCCAGAAAACAGGCAC	MKFLVNVALVFMVVYISYIYADPGHHHHHHGTAENLYFQGSRTDPLVLVFVESLYSQLGQEVVA<br>ILESSRFKYRTEIAPGKGDMPTLTDKGRGRFALIIYENILKYVNLDAWNRELLDKYCVAYGVGI<br>IGFFKANENSLLSAQLKGFPLFLHSNLGLKDCSINPKSPLLYVTRPSEVEKGVLPGEDWTVFQS<br>NHSTYEPVLLAKTRSSESIPHLGADAGLHAALHATVVQDLGLHDGIQRVLFGNNLNFWLHKLVF<br>VDAVAFLTGKRLSLPLDRYILVDIDDIFVGKEGTRMKVEDVKALFDTQNELRAHIPNFTFNLGY<br>SGKFFHTGTNAEDAGDDLLLSYVKEFWWFPHMWSHMQPHLFHNQSVLAEQMALNKKFAVEHGIP<br>TDMGYAVAPHHSGVYPVHVQLYEAWKQVWSIRVTSTEEYPHLKPARYRRGFIHNGIMVLPRQTC<br>GLFTHTIFYNEYPGGSSELDKIINGGELFLTVLLNPISIFMTALSNYGNDRLGLYTFKHLVRFL<br>HSWTNLRLQTLPPVQLAQKYFQIFSEEKDPLWQDPCEDKRHKDIWSKEKTCDRFPKLLIIGPQK<br>TGTTALYLFLGMHPDLSSNYPSSETFEEIQFFNGHNYHKGIDWYMEFFPIPSNTTSDFYFEKSA<br>NYFDSEVAPRRAAALLPKAKVLTILINPADRAYSWYQHQRAHDDPVALKYTFHEVITAGSDASS<br>KLRALQNRCLVPGWYATHIERWLSAYHANQILVLDGKLLRTEPAKVMDMVQKFLGVTNTIDYHK<br>TLAFDPKKGFWCQLLEGGKTKCLGKSKGRKYPEMDLDSRAFLKDYYRDHNIELSKLLYKMGQTL<br>PTWLREDLQNTR*

	CACTGCCCTCTACCTGTTCCTGGGCATGCACCCTGACCTAAGCAGCAACTACCCCAGCTCTG<br>AGACATTTGAGGAGATCCAGTTTTTTAATGGCCACAACTATCACAAAGGCATCGACTGGTAC<br>ATGGAGTTCTTCCCCATCCCTTCCAACACCACGTCCGACTTCTACTTTGAGAAAAGCGCCAA<br>CTACTTTGATTCAGAAGTGGCGCCCCGGCGGGCAGCAGCCCTCTTGCCCAAAGCCAAGGTCC<br>TGACCATCCTCATCAACCCCGCGGACCGGGCCTATTCCTGGTACCAGCACCAGCGAGCCCAT<br>GACGACCCAGTGGCCCTAAAGTACACCTTCCATGAGGTGATTACCGCCGGCTCTGACGCATC<br>CTCGAAGCTGCGTGCCCTCCAGAACCGCTGCCTGGTCCCTGGCTGGTACGCCACCCACATCG<br>AGCGCTGGCTCAGTGCCTATCACGCCAACCAGATTCTGGTCTTGGATGGCAAACTGCTTCGC<br>ACAGAACCTGCCAAAGTGATGGACATGGTGCAGAAGTTCCTTGGGGTGACCAACACCATTGA<br>CTACCACAAAACCTTGGCGTTTGATCCAAAGAAAGGATTTTGGTGCCAACTGCTTGAAGGAG<br>GAAAAACCAAGTGTCTGGGCAAAAGCAAGGGCCGGAAATATCCCGAGATGGACTTGGATTCC<br>CGAGCCTTCCTGAAGGACTATTACCGGGACCACAACATCGAGCTCTCCAAGCTGCTGTATAA<br>GATGGGCCAGACACTTCCCACTTGGCTACGAGAGGACCTCCAGAACACCAGGTAA	
NDST ST domain (602–882)	ATGAAATTTTTGGTGAACGTGGCCTTGGTGTTTATGGTGGTTTACATTAGCTACATTTATGC<br>GGACCCGGGCCATCATCACCACCATCATGGTACCGCAGAAAACTTGTACTTTCAAGGCGACC<br>GCTTCCCAAAGCTCCTCATCATCGGCCCCCAGAAAACAGGCACCACTGCCCTCTACCTGTTC<br>CTGGGCATGCACCCTGACCTAAGCAGCAACTACCCCAGCTCTGAGACATTTGAGGAGATCCA<br>GTTTTTTAATGGCCACAACTATCACAAAGGCATCGACTGGTACATGGAGTTCTTCCCCATCC<br>CTTCCAACACCACGTCCGACTTCTACTTTGAGAAAAGCGCCAACTACTTTGATTCAGAAGTG<br>GCGCCCCGGCGGGCAGCAGCCCTCTTGCCCAAAGCCAAGGTCCTGACCATCCTCATCAACCC<br>CGCGGACCGGGCCTATTCCTGGTACCAGCACCAGCGAGCCCATGACGACCCAGTGGCCCTAA<br>AGTACACCTTCCATGAGGTGATTACCGCCGGCTCTGACGCATCCTCGAAGCTGCGTGCCCTC<br>CAGAACCGCTGCCTGGTCCCTGGCTGGTACGCCACCCACATCGAGCGCTGGCTCAGTGCCTA<br>TCACGCCAACCAGATTCTGGTCTTGGATGGCAAACTGCTTCGCACAGAACCTGCCAAAGTGA<br>TGGACATGGTGCAGAAGTTCCTTGGGGTGACCAACACCATTGACTACCACAAAACCTTGGCG<br>TTTGATCCAAAGAAAGGATTTTGGTGCCAACTGCTTGAAGGAGGAAAAACCAAGTGTCTGGG<br>CAAAAGCAAGGGCCGGAAATATCCCGAGATGGACTTGGATTCCCGAGCCTTCCTGAAGGACT<br>ATTACCGGGACCACAACATCGAGCTCTCCAAGCTGCTGTATAAGATGGGCCAGACACTTCCC<br>ACTTGGCTACGAGAGGACCTCCAGAACACCAGGTAA	MKFLVNVALVFMVVYISYIYADPGHHHHHHGTAENLYFQGDRFPKLLIIGPQKTGTTALYLFLG<br>MHPDLSSNYPSSETFEEIQFFNGHNYHKGIDWYMEFFPIPSNTTSDFYFEKSANYFDSEVAPRR<br>AAALLPKAKVLTILINPADRAYSWYQHQRAHDDPVALKYTFHEVITAGSDASSKLRALQNRCLV<br>PGWYATHIERWLSAYHANQILVLDGKLLRTEPAKVMDMVQKFLGVTNTIDYHKTLAFDPKKGFW<br>CQLLEGGKTKCLGKSKGRKYPEMDLDSRAFLKDYYRDHNIELSKLLYKMGQTLPTWLREDLQNT<br>R*
nAb1	ATGAAATACCTATTGCCTACGGCGGCCGCTGGATTGTTATTACTCGCGGCCCAGCCGGCCAT<br>GGCCCAGGTGCAGCTGGTCGAGTCTGGGGGAGGATTGGTGCAGGCTGGGGGCTCTCTGAGAC<br>TCTCCTGTGCAGCCTCTGACCGCACCTTCACTACTTATGCCATGGCCTGGTTCCGCCGGGCT<br>CCTCCAGGGAAGGAGCGTGAGTTTGTAGCCTCTATTAGGTGGGATGGTTTTGGTACATACTA<br>TACTGACTCCGTGAAGGGCCGATTCACCATCTCCAGAGACAGCGCCAAGAACGAGGCTTACC<br>TGCAAATGAACAGCCTGAGACCTGAGGACACGGGCGTTTATTACTGTGCAGCAGGCTTACTG<br>TTCGGTAACACTAAAGGTTATGACCACTGGGGCCAGGGGACCCAGGTCACCGTCTCCTCAGA<br>ACCCAAGACACCAAAACCACAACCAGCGGCCGGCCCGGGAGGCCAACACCATCACCACCATC<br>ATGGCGCAGAACAAAAACTCATCTCAGAAGAGGATCTGTCTTAG	MKYLLPTAAAGLLLLAAQPAMAQVQLVESGGGLVQAGGSLRLSCAASDRTFTTYAMAWFRRAPP<br>GKEREFVASIRWDGFGTYYTDSVKGRFTISRDSAKNEAYLQMNSLRPEDTGVYYCAAGLLFGNT<br>KGYDHWGQGTQVTVSSEPKTPKPQPAAGPGGQHHHHHHGAEQKLISEEDLS*
nAb5	ATGAAATACCTATTGCCTACGGCGGCCGCTGGATTGTTATTACTCGCGGCCCAGCCGGCCAT<br>GGCCCAGGTGCAGCTGGTCGAGTCTGGGGGAGGCTTGGTGCAGGCTGGGGGCTCTCTGAGAC<br>TCTCCTGTGCAGCCTCTGGACGCACCTTCAGTAGCTATGTCATGGGCTGGTTCCGCCAGGCT<br>CCAGGGAAGGAGCGTGAGTTTGTAGCAGCTATTAGCTGGAGTGGTGGTACGACAGACTATGC<br>AGGCTCCGTGAAGGGCCGATTCACCATCTCCAGAGACAATGACAGGAAGACCATGTATCTAG<br>AGATGAACGACCTGAAGCCTGAGGACACGGCCCTTTACTACTGCAATTTTAAGGAGACTTGG<br>CCCCGAACGGGCGACTACTGGGGACCGGGGACCCAGGTCACCGTCTCCTCAGAACCCAAGAC<br>ACCAAAACCACAACCAGCGGCCGGCCCGGGAGGCCAACACCATCACCACCATCATGGCGCAG<br>AACAAAAACTCATCTCAGAAGAGGATCTGTCTTAG	MKYLLPTAAAGLLLLAAQPAMAQVQLVESGGGLVQAGGSLRLSCAASGRTFSSYVMGWFRQAPG<br>KEREFVAAISWSGGTTDYAGSVKGRFTISRDNDRKTMYLEMNDLKPEDTALYYCNFKETWPRTG<br>DYWGPGTQVTVSSEPKTPKPQPAAGPGGQHHHHHHGAEQKLISEEDLS*
nAb6	ATGAAATACCTATTGCCTACGGCGGCCGCTGGATTGTTATTACTCGCGGCCCAGCCGGCCAT<br>GGCCCAGGTGCAGCTGGTCGAGTCTGGGGGAGACTTGGTGCAGCCTGGGGGATCTCTGAGAC<br>TCTCCTGTGCAGCCTCGAAAACCATCTTCGTTATCAGTGACATGGGTTGGTACCGCCAGGCT<br>CCAGGAAAGCAGCGCGAGTTGGTCGCAATGATTACTAGTGGTGGTACCACAAACTATGCAGA<br>CTCTGTGAAGGGCCGATTCACCATCTCCAGAGACAACACCCAGAACACGGTGTGGCTGCAGA<br>TGAACAGCCTGAAACCTGAGGACACGGCCGTTTATTATTGCTATGCGCGCGTGGAACGTTGG<br>AGGCCTACTGACTCCTGGGGCCAGGGGACCCAGGTCACCGTCTCCTCAGAACCCAAGACACC<br>AAAACCACAACCAGCGGCCGGCCCGGGAGGCCAACACCATCACCACCATCATGGCGCAGAAC<br>AAAAACTCATCTCAGAAGAGGATCTGTCTTAG	MKYLLPTAAAGLLLLAAQPAMAQVQLVESGGDLVQPGGSLRLSCAASKTIFVISDMGWYRQAPG<br>KQRELVAMITSGGTTNYADSVKGRFTISRDNTQNTVWLQMNSLKPEDTAVYYCYARVERWRPTD<br>SWGQGTQVTVSSEPKTPKPQPAAGPGGQHHHHHHGAEQKLISEEDLS*

nAb7	ATGAAATACCTATTGCCTACGGCGGCCGCTGGATTGTTATTACTCGCGGCCCAGCCGGCCAT<br>GGCCCAGGTGCAGCTGGTCGAGTCTGGAGGAGGCTCGGTGCAGGCTGGGGGGTCTCTGAGAC<br>TCTCCTGTGCAGCCTCTGGATTCAATGTGGATGATTATGCTATAGGCTGGTTCCGCCAGTCT<br>CCGGGGAAGGAGCGTGAGGGGGTCTCATGTATTGGCGGTGATGGTACCACATATTATGAAAA<br>TTCCGTGAAGGGCCGATTCACCGTCTCCAGTGACAAGCGAGACAACACGGTGTATCTGCAAA<br>TGAACAACCTGAGACCTGAGGACACGGCCATTTACTTCTGTGCAGCAGATCGGTCGAAATAC<br>TGTGTTGGTAAATACTTCTCGACGCCTTCTCAATATGACTTCTGGGGCCGGGGCACCCACGT<br>CACCGTCTCTTCAGAACCCAAGACACCAAAACCACAACCAGCGGCCGGCCCGGGAGGCCAAC<br>ACCATCACCACCATCATGGCGCAGAACAAAAACTCATCTCAGAAGAGGATCTGTCTTAG	MKYLLPTAAAGLLLLAAQPAMAQVQLVESGGGSVQAGGSLRLSCAASGFNVDDYAIGWFRQSPG<br>KEREGVSCIGGDGTTYYENSVKGRFTVSSDKRDNTVYLQMNNLRPEDTAIYFCAADRSKYCVGK<br>YFSTPSQYDFWGRGTHVTVSSEPKTPKPQPAAGPGGQHHHHHHGAEQKLISEEDLS*
nAb13	ATGAAATACCTATTGCCTACGGCGGCCGCTGGATTGTTATTACTCGCGGCCCAGCCGGCCAT<br>GGCCCAGGTGCAGCTGGTCGAGTCTGGGGGAGGATTGGTGCAGTCCGGGGGCTCTCTGAGAC<br>TCTCCTGTACAGCCTCAAGACGCGCCTCTTCCACCATAGGCTGGTTCCGCCAGGCTCCAGGG<br>AAGGAGCGTGAGTTTGTGGCCGGTATTAATTGGAGTCTGGAAACGGCAACCTATCAAGACTC<br>CATTAAGGATCGATTCACCATCTCCAGAGACAACGCCAACAACACGGTGTACTTGCAAATGA<br>ACAGCCTGAAACCTGAGGACACGGCCGTTTATTACTGTGCAGCGTCCCCTTATCGGGTAATG<br>GGTGACACCACACTGTATCCATACTGGGGCCAGGGGACCCAGGTCACCGTCTCCTCAGAACC<br>CAAGACACCAAAACCACAACCAGCGGCCGGCCCGGGAGGCCAACACCATCACCACCATCATG<br>GCGCAGAACAAAAACTCATCTCAGAAGAGGATCTGTCTTAG	MKYLLPTAAAGLLLLAAQPAMAQVQLVESGGGLVQSGGSLRLSCTASRRASSTIGWFRQAPGKE<br>REFVAGINWSLETATYQDSIKDRFTISRDNANNTVYLQMNSLKPEDTAVYYCAASPYRVMGDTT<br>LYPYWGQGTQVTVSSEPKTPKPQPAAGPGGQHHHHHHGAEQKLISEEDLS*

Signal peptides are highlighted in cyan. Tags are highlighted in green. Protease cleavage sites are highlighted in pink.

Supplementary Table 3 Primers used for cloning and mutagenesis of NDST1 and SULT1A1

Primer	Sequence
NDST1 F	cttgtactttcaaggcTCCCGCACAGACCCGTTGGTG
NDST1 R	cttctcgacaagcttTTACCTGGTGTTCTGGAGGTCCTC
NDST1 C-Avitag R	cttctcgacaagcttTTACTCGTGCCACTCGATTTTCTGGGCCTCGAAGATGTCGTTCAGGCCtccgcttgaCCTGGTGTTCTGGAGGTCC
NDST1 D319A mutagenesis F	GGACATTgcgGACATCTTCGTGGGCAAGGAGGG
NDST1 D319A mutagenesis R	CGAAGATGTCcgcAATGTCCACCAGGATGTAGCGG
NDST1 D320A mutagenesis F	GGACATTGATgcgATCTTCGTGGGCAAGGAGGGCAC
NDST1 D320A mutagenesis R	CCACGAAGATcgcATCAATGTCCACCAGGATGTAGCGG
NDST1 H389A mutagenesis F	GGTTCCCCgcgATGTGGAGCCACATGCAGCCCC
NDST1 H389A mutagenesis R	GGCTCCACATcgcGGGGAACCACCAGAACTCCTTC
NDST1 H393A mutagenesis F	ATGTGGAGCgcgATGCAGCCCCACCTTTTCCACAACCAG
NDST1 H393A mutagenesis R	GGGGCTGCATcgcGCTCCACATGTGGGGGAACCACC
NDST1 H529A mutagenesis F	TCATGACGgcgCTGTCCAACTATGGGAATGACCG
NDST1 H529A mutagenesis R	TAGTTGGACAGcgcCGTCATGAAGATGCTGATAGGATTG
SULT1A1 F	gttcacatATGGAGTTCTCCCGTCCACC
SULT1A1 R	gatcggatccTCATAGTTCACAACGAAACTTG
SULT1A1 K65E;R68G mutagenesis F	CTAGAGgaaTGTGGCggtGCCCCCATCTATGCCCGG
SULT1A1 K65E;R68G mutagenesis R	GGGGGCACCGCCACATTCCTCTAGCTTGCCACCCTG

Supplementary references

1. Sievers, F. et al. Fast, scalable generation of high-quality protein multiple sequence alignments using Clustal Omega. *Mol Syst Biol* **7**, 539 (2011).
2. Vallet, S.D. et al. Functional and structural insights into human N-deacetylase/N-sulfotransferase activities. *Proteoglycan Research* **1**, e8 (2023).
