# Supplementary material for: Structural and mechanistic characterization of bifunctional heparan sulfate N-deacetylase-N-sulfotransferase 1
Source: Nat Commun. 2024 Feb 13;15:1326. doi: 10.1038/s41467-024-45419-4 (PMC10864358; doi:10.1038/s41467-024-45419-4)
Supplement: Supplementary file 3 — Description of Additional Supplementary Files [file 41467_2024_45419_MOESM3_ESM.pdf]

File name: Supplementary Movie 1
Description: Breathing motion for NDST1-nAb7 complex as described by cryoSPARC 3D variability analysis

File name: Supplementary Movie 2
Description: Breathing motion for NDST1-nAb13 complex as described by cryoSPARC 3D variability analysis
