# Supplementary material for: Structural and mechanistic characterization of bifunctional heparan sulfate N-deacetylase-N-sulfotransferase 1
Source: Nat Commun. 2024 Feb 13;15:1326. doi: 10.1038/s41467-024-45419-4 (PMC10864358; doi:10.1038/s41467-024-45419-4)
Supplement: Supplementary file 6 — Reporting Summary [file 41467_2024_45419_MOESM6_ESM.pdf]

nature portfolio

Corresponding author(s): Liang Wu

Last updated by author(s): Oct 30, 2023

Reporting Summary

Nature Portfolio wishes to improve the reproducibility of the work that we publish. This form provides structure for consistency and transparency in reporting. For further information on Nature Portfolio policies, see our Editorial Policies and the Editorial Policy Checklist.

Statistics

For all statistical analyses, confirm that the following items are present in the figure legend, table legend, main text, or Methods section.

n/a	Confirmed	
☐	☒	The exact sample size (n) for each experimental group/condition, given as a discrete number and unit of measurement
☐	☒	A statement on whether measurements were taken from distinct samples or whether the same sample was measured repeatedly
☒	☐	The statistical test(s) used AND whether they are one- or two-sided<br>*Only common tests should be described solely by name; describe more complex techniques in the Methods section.*
☒	☐	A description of all covariates tested
☒	☐	A description of any assumptions or corrections, such as tests of normality and adjustment for multiple comparisons
☐	☒	A full description of the statistical parameters including central tendency (e.g. means) or other basic estimates (e.g. regression coefficient) AND variation (e.g. standard deviation) or associated estimates of uncertainty (e.g. confidence intervals)
☒	☐	For null hypothesis testing, the test statistic (e.g. F, t, r) with confidence intervals, effect sizes, degrees of freedom and P value noted<br>*Give P values as exact values whenever suitable.*
☒	☐	For Bayesian analysis, information on the choice of priors and Markov chain Monte Carlo settings
☒	☐	For hierarchical and complex designs, identification of the appropriate level for tests and full reporting of outcomes
☒	☐	Estimates of effect sizes (e.g. Cohen's d, Pearson's r), indicating how they were calculated

Our web collection on statistics for biologists contains articles on many of the points above.

Software and code

Policy information about availability of computer code

Data collection	Clariostar software version 5.70 R2 (BMG Labtech)<br>Octet BLI discovery version 12.2.2.20 (Sartorius)<br>QuantStudio Design and Analysis Software v1.5.2 (Applied Biosystems)<br>Biacore T200 control software version 2.0.2 (GE Healthcare)<br>EPU version 3.2.0 (Thermo Fisher Scientific)<br>Chirascan control 2.02 (Applied Photophysics)
Data analysis	Octet analysis studio version 12.2.2.26 (Sartorius)<br>Protein Thermal Shift Software v1.4 (Applied Biosystems)<br>Biacore T200 evaluation software version 3.1 (GE Healthcare)<br>Cryosparc 2.14.1<br>Relion 3.1.1<br>Prism 10.1.0 (Graphpad)

For manuscripts utilizing custom algorithms or software that are central to the research but not yet described in published literature, software must be made available to editors and reviewers. We strongly encourage code deposition in a community repository (e.g. GitHub). See the Nature Portfolio guidelines for submitting code & software for further information.

Data

Policy information about availability of data

All manuscripts must include a data availability statement. This statement should provide the following information, where applicable:

- Accession codes, unique identifiers, or web links for publicly available datasets
- A description of any restrictions on data availability
- For clinical datasets or third party data, please ensure that the statement adheres to our policy

The cryo-EM data generated in this study have been deposited in the PDB and EMDB under accession codes 8CCY (NDST1 alone model), EMD-16564 (NDST1 alone map), 8CD0 (NDST1-nAb7 model), EMD-16627 (NDST1-nAb7 NTD and deacetylase domain local map), EMD-16629 (NDST1-nAb7 deacetylase and sulfotransferase domain local map), EMD-16565 (NDST1-nAb7 composite map), EMD-16626 (NDST1-nAb7 original map), 8CHS (NDST1-nAb13 model), EMD-16662 (NDST1-nAb13 deacetylase and sulfotransferase domain local map), EMD-16663 (NDST1-nAb13 deacetylase and sulfotransferase domain local map), EMD-16664 (NDST1-nAb13 composite map), EMD-16661 (NDST1-nAb13 original map). We have also referenced in this work PDB accessions 6NWZ (Agd3 deacetylase), 3UAN (HS3ST1), 6XL8 (HS3ST3), and EMDB accession EMD-17349 (NDST1 dimer). DNA and protein sequences for NDST1 and nAb constructs are available in Supplementary Table 2. Source data are provided with this paper.

Research involving human participants, their data, or biological material

Policy information about studies with human participants or human data. See also policy information about sex, gender (identity/presentation), and sexual orientation and race, ethnicity and racism.

Reporting on sex and gender	N/A
Reporting on race, ethnicity, or other socially relevant groupings	N/A
Population characteristics	N/A
Recruitment	N/A
Ethics oversight	N/A

Note that full information on the approval of the study protocol must also be provided in the manuscript.

Field-specific reporting

Please select the one below that is the best fit for your research. If you are not sure, read the appropriate sections before making your selection.

☒ Life sciences ☐ Behavioural & social sciences ☐ Ecological, evolutionary & environmental sciences

For a reference copy of the document with all sections, see nature.com/documents/nr-reporting-summary-flat.pdf

Life sciences study design

All studies must disclose on these points even when the disclosure is negative.

Sample size	Assay datapoints were collected in technical triplicate or quadruplicate. The chosen sample sizes are standard for small scale biochemical experiments, where differences between treatment and control conditions are relatively unambiguous.
Data exclusions	A few technical replicates in assays were excluded where clear experimental artifacts were apparent (e.g. bubbles in microplate wells).<br><br>Cryo-EM micrographs were excluded based on the quality of ice, as judged from CTF information, or evaluation of excessive motion (max 26 pix total) as calculated by cryoSPARC patch motion correction. Based on these criteria, typically ~15% of micrographs were excluded from the initially collected data.
Replication	Assay datapoints were collected in technical triplicate or quadruplicate. Results between replicates were consistent, except for a few instances where clear experimental artifacts were apparent (e.g. bubbles in wells).
Randomization	Small scale biochemical assays - no randomization practicable.
Blinding	Small scale biochemical assays - no blinding practicable.

Reporting for specific materials, systems and methods

We require information from authors about some types of materials, experimental systems and methods used in many studies. Here, indicate whether each material, system or method listed is relevant to your study. If you are not sure if a list item applies to your research, read the appropriate section before selecting a response.

Materials & experimental systems

n/a	Involved in the study
☐	☒ Antibodies
☐	☒ Eukaryotic cell lines
☒	☐ Palaeontology and archaeology
☒	☐ Animals and other organisms
☒	☐ Clinical data
☒	☐ Dual use research of concern
☒	☐ Plants

Methods

n/a	Involved in the study
☒	☐ ChIP-seq
☒	☐ Flow cytometry
☒	☐ MRI-based neuroimaging

Antibodies

Antibodies used	New nanobodies against NDST1 were developed as part of this work. Llama immunizations were approved under project license PA1FB163A (University of Reading, UK).<br><br>HRP streptavidin (Thermo 21124) and HRP-anti-M13 (Cytiva 27-9421-01)
Validation	Binding studies (SPR, BLI) against NDST1 are described herein. Structural complexes were determined for 2 of the nanobodies.<br><br>HRP streptavidin (Thermo 21124) and mouse HRP-anti-M13 (Cytiva 27-9421-01) were used from the supplier without further validation.<br><br>- For 21124 - manufacturer validated binding activity to be at least 7 ug Biotin bound/mg protein. (https://www.thermofisher.com/document-connect/document-connect.html?url=https://assets.thermofisher.com/TFS-Assets%2FLSG%2Fcertificate%2FCertificates-of-Analysis%2F21126_YG377723.PDF)<br>- For 27-9421-01 - manufacturer validated binding of at least 0.5E9 pfu/ml of M13 phage captured by bound anti-M13 Monoclonal Antibody with an A405 >0.6 using ABTS substrate. (https://uk.vwr.com/assetsvc/asset/en_GB/id/9458946/contents)

Eukaryotic cell lines

Policy information about cell lines and Sex and Gender in Research

Cell line source(s)	High Five (Trichoplusia ni) and ExpiSf9 (Spodoptera frugiperda) cells - both Thermo Fisher
Authentication	Used from supplier without further authentication
Mycoplasma contamination	Tested negative for mycoplasma
Commonly misidentified lines (See ICLAC register)	None
